# Pan-plantae aquaporin evolution: insights into *PIP2* as a potential regulator of rose prickle development

**DOI:** 10.1093/hr/uhag143

**Published:** 2026-03-14

**Authors:** Hong Zeng, Shi-Zhao Zhou, Hai-Ling Zhang, Bin-Yan Zhao, Yi-Liang Liu, Yan-Hong Zeng, Xiao-Dong Jiang, Wei-Hua Cui, Xue Dong, Jin-Yong Hu, Mi-Cai Zhong

**Affiliations:** State Key Laboratory of Plant Diversity and Specialty Crops, Yunnan Key Laboratory of Crop Wild Relatives Omics, Kunming Institute of Botany, Chinese Academy of Sciences, Kunming 650201, Yunnan, China; Kunming College of Life Science, University of Chinese Academy of Sciences, Kunming 650204, Yunnan, China; State Key Laboratory of Plant Diversity and Specialty Crops, Yunnan Key Laboratory of Crop Wild Relatives Omics, Kunming Institute of Botany, Chinese Academy of Sciences, Kunming 650201, Yunnan, China; Kunming College of Life Science, University of Chinese Academy of Sciences, Kunming 650204, Yunnan, China; State Key Laboratory of Plant Diversity and Specialty Crops, Yunnan Key Laboratory of Crop Wild Relatives Omics, Kunming Institute of Botany, Chinese Academy of Sciences, Kunming 650201, Yunnan, China; Kunming College of Life Science, University of Chinese Academy of Sciences, Kunming 650204, Yunnan, China; State Key Laboratory of Plant Diversity and Specialty Crops, Yunnan Key Laboratory of Crop Wild Relatives Omics, Kunming Institute of Botany, Chinese Academy of Sciences, Kunming 650201, Yunnan, China; Kunming College of Life Science, University of Chinese Academy of Sciences, Kunming 650204, Yunnan, China; State Key Laboratory of Plant Diversity and Specialty Crops, Yunnan Key Laboratory of Crop Wild Relatives Omics, Kunming Institute of Botany, Chinese Academy of Sciences, Kunming 650201, Yunnan, China; Kunming College of Life Science, University of Chinese Academy of Sciences, Kunming 650204, Yunnan, China; College of Horticulture Science and Engineering & Center for Interdisciplinary Biodiversity Research, Shandong Agricultural University, Tai' An 271018, Shandong, China; College of Horticulture Science and Engineering & Center for Interdisciplinary Biodiversity Research, Shandong Agricultural University, Tai' An 271018, Shandong, China; College of Forestry & Center for Interdisciplinary Biodiversity Research, Shandong Agricultural University, Tai' An 271018, Shandong, China; College of Horticulture Science and Engineering & Center for Interdisciplinary Biodiversity Research, Shandong Agricultural University, Tai' An 271018, Shandong, China; State Key Laboratory of Plant Diversity and Specialty Crops, Yunnan Key Laboratory of Crop Wild Relatives Omics, Kunming Institute of Botany, Chinese Academy of Sciences, Kunming 650201, Yunnan, China; College of Horticulture Science and Engineering & Center for Interdisciplinary Biodiversity Research, Shandong Agricultural University, Tai' An 271018, Shandong, China

## Abstract

Plant aquaporin (AQP), one of the ancient protein superfamilies with high diversity, plays a canonical role as water channel transporters to regulate seed germination, stomatal movement, cell elongation, reproduction, and environmental adaptation. Previously, we have demonstrated that two *AQP*s colocalize within a quantitative trait locus (QTL) linked to the genetic regulation of rose prickles, which function as water reservoirs at their early developmental stages. Here, we systematically characterize the diversity of AQPs in all plant lineages taking the Rosaceae as one example and establish a genetic link between *PIP2;1* expression and prickle development. We reveal the ancient origin and remarkable deep conservation in AQP protein structure, the increase of copy number following the diversification of plant lineages and a strong positive selection of the *PIP2* and the *SIP* subfamilies within Rosaceae. Intriguingly, this is contrasted by substantial expression divergence, which is accompanied with regulatory divergence, particularly in *cis-*regulatory elements associated with hormone responses and developmental regulation. Spatial–temporal expression profiling demonstrates dynamic *AQP* expression patterns during development of prickle and the underlying cortical tissue in the prickly *Rosa gigantea*, while also differ substantially in shoots and leaves of the prickly *Rosa chinensis* and prickle-free *Rosa wichuraiana*. Silencing the expression of *PIP2;1* within the prickle QTL region significantly attenuates the prickle production along rose stems. Our findings not only highlight both evolutionary conservation in AQP protein architecture and lineage-specific regulatory innovation in Rosaceae but also uncover a novel regulatory role of *PIP2;1* in epidermal differentiation of the non-model roses, and of likely many other spiny angiosperms.

## Highlights

Systematic and genome-wide characterization of plantae aquaporins reveals a high conservation in protein sequences but accompanied by remarkable regulatory sequence diversity. Genetic analysis demonstrates that *PIP2* regulates potentially the prickle development in rose, thus providing fresh insights into aquaporin function and molecular regulators involved in prickle patterning.

## Introduction

Aquaporins (AQPs), a class of integral membrane proteins in the major intrinsic protein superfamily, serve as critical regulators of transmembrane water flux and cellular homeostasis in plants. AQPs are phylogenetically classified into five subfamilies, PIPs (Plasma membrane Intrinsic Proteins), TIPs (Tonoplast Intrinsic Proteins), NIPs (NOD26-like Intrinsic Proteins), small basic intrinsic proteins (SIPs), and uncategorized intrinsic proteins (XIPs) (Tables S5) [1, 2]. Besides water transportation, emerging evidence has identified the pleiotropic roles of AQPs in plant physiology, including transpiration, nutrient transport, and responses to abiotic stresses such as drought and salinity [3–9]. Arabidopsis *PIP1;2* and *PIP2;2* are critical for maintaining protoplast water permeability [4, 10]. Corroborating with their expression variation upon drought stress, overexpression of some *AQP* genes like *TIP2* and *TIP4* improves drought tolerance in transgenic plants [9, 11]. AQPs also facilitate the transport of other solutes like urea, ammonia, and hydrogen peroxide, which are vital for nutrient uptake and plant health [7, 12–16]. Interaction of AQPs with mycorrhizal fungi improves nutrient uptake and drought tolerance [5, 17]. Phosphorylation of PIP2;1 by CPK12 enhances reactive oxygen species (ROS) transport, thereby increasing plant disease resistance [16]. Plant AQPs are important for organ development. In *Arabidopsis*, organ-specific expression of *TIP1;1* in roots and aerial tissues coordinates cell expansion through vacuolar membrane dynamics [18]. In the developmental programs, AQPs often interact with phytohormones, such as the auxin-mediated regulation of *PIP* splicing governing lateral root primordia emergence via auxin response factors and the gibberellin-induced *TIP1;1* expression promoting vacuolar expansion and root elongation [19, 20]. In the nonmodel species like roses (*Rosa* spp.), AQP function extends to petal morphogenesis, where ethylene-responsive *RhPIP1;1-RhPIP2;1* interaction modulates cellular water influx during flower opening [21–26]. Additionally, AQPs also play a role in plant defenses to pathogen challenge, thus highlighting their dual functionality in balancing growth and defense trade-offs [27].

Plant prickles, which are sometimes considered as modified trichomes, are appendage structures, but without vasculature contact, on the surfaces of stems, leaves, or other tissues of many angiosperms including, roses, one of the most important ornamental plants worldwide [2]. While trichome development has been extensively characterized in model plants [28, 29], the molecular basis of prickle formation in roses remains enigmatic. Most roses contain prickles on their stems. Morphological and anatomical analyses have revealed that their ontogenic origins are different, with trichomes generated from epidermal growth and prickles meristemed from cortical cells below the surface, hence under control by different gene regulatory networks (GRNs) [30]. Not surprisingly, the homologs of Arabidopsis genes involved in trichome initiation are not always transcriptionally regulated during the prickle development [31]. However, heterologous expression of rose *CAPRICE* leads to reduced trichome production in Arabidopsis, suggesting that some hubs of the trichome-related GRN might be functionally conserved [32]. Indeed, repeated natural mutations in a cytokinin biosynthetic gene *LONELY GUY 3* (*LOG3*) have convergently led to a frequent prickle-free pattern in *Solanum* and roses as well as many other plant lineages [33]. A comparative transcriptomics analysis reveals a complex prickle-related GRN coupled to secondary metabolism transcription factors in *Rosa multiflora* [34]. Despite these, the molecular and genetic mechanisms underlying prickle development remain poorly explored.

Besides its roles in defenses and preventing water loss, rose prickles can serve as water reservoirs at their early developmental stages [35]. Physiological analyses reveal a high relative water content (RWC) to ~84% in developing prickles compared to adjacent cortical tissues (~76%), while the RWC can reach ~20% when prickles are fully lignified. Intriguingly, two *PIP2*s collocate within the quantitative trait locus 1 (QTL1), a region enriching genes involved in water usage and desiccation responses, and feature highly diverged expression patterns between the prickly *R. chinensis* `Old Blush' (OB) and the prickle-free *R. wichuraiana* `Basye's Thornless' (BT) [35].

In this study, we systematically characterized *AQP*s' evolutionary pattern along major lineages of the plant kingdom and at the family level by focusing on the biologically and economically important Rosaceae family. We showed that their protein coding sequences are remarkably conserved, while both *PIP2* and *SIP* subfamilies were under pressure of positive selection. We identified a spatial–temporal expression pattern for *AQP*s, which featured a distinct coexpression network with genes involved in plant cell wall establishment and was substantiated with substantial regulatory divergences particularly prevalent in *cis-*regulatory elements associated with hormone responses and developmental regulation. We functionally justified the prominent roles of *PIP2;1* in rose prickle development via virus-induced gene silencing (VIGS) and RNA-seq approaches. Our efforts unveil a highly diversified evolutionary pattern of plantae AQPs, establishing the *PIP2* as potential regulators of prickle development, thus providing fresh insights into the genetic control of prickle patterning in woody perennials.

## Results

### AQP proteins are shared across all major lineages of the plant kingdom

**Figure 1:**
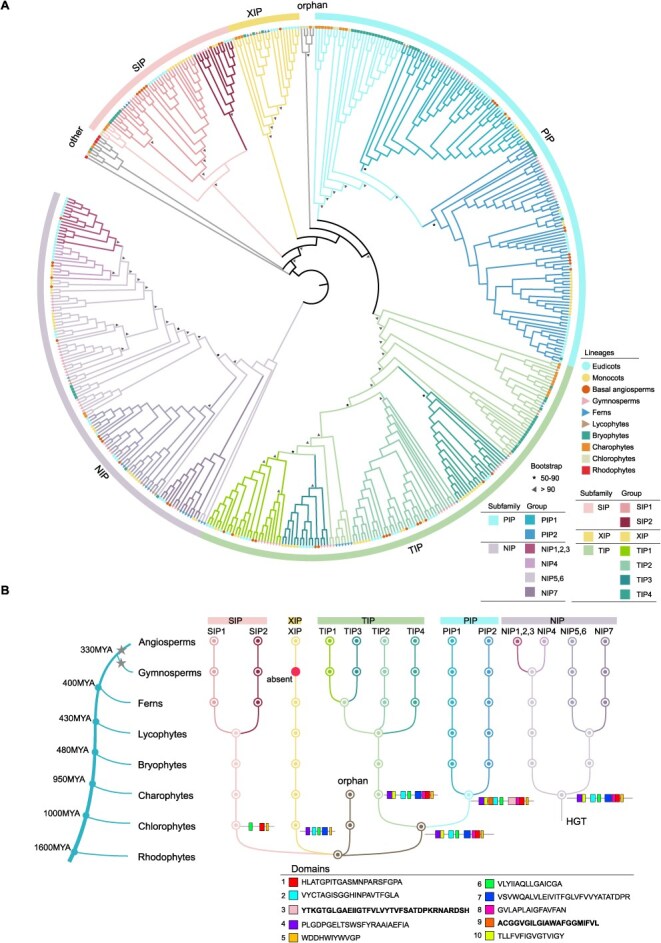
A deep conservation of AQP subfamilies in major lineages of plant kingdom. (A) Phylogenetic clustering of 524 AQPs using an unrooted maximum likelihood method with bootstrap supports for major clusters for 20 selected genomes representing all major lineages of the plant kingdom. The * and triangle indicate bootstrap supports of 50%–90% and >90%, respectively. Five major subfamilies are marked in distinct colors: SIP, XIP, PIP, TIP, and NIP. Internal groups within each subfamily are further distinguished by shades of its assigned color. Different label shapes with distinct color represent the major lineages of the plant kingdom. `Orphan' marks the Chlorophyte and Charophyte genes clustering together but not belonging to any of the PIP, TIP, and NIP subfamilies. `Other' labels the genes not belonging to any of the five major subfamilies. The AQPs clustered within certain subfamilies but unable to be assigned to known groups were colored according to the subfamily's color scheme. (B) A summarized evolutionary dynamics for AQP subfamilies in major lineages of plant kingdom. Phylogeny of taxonomic classes are shown in left and numbers are estimated time of divergence expressed as millions of years ago (MYA). The stars mark the two whole genome duplication events along angiosperms and gymnosperms lineages, respectively. The simplified phylogeny shows the evolutionary relationships of each subfamily in the common ancestors along lineages. Circles are colored for groups of each subfamily corresponding to (A). Top row represents the AQP subfamily with names showing the genes from *A. thaliana*. Cartoons close to early lineages show the predicted protein domain or motifs for each subfamily. XIP is not found in gymnosperms. The two domains with bold sequences give the motifs specific for the PIP subfamily proteins. `HGT' means Horizontal Gene Transfer.

The genome of the flowering plant *Arabidopsis thaliana* encodes 38 AQP proteins, including 3 SIPs, 11 TIPs, 14 PIPs, and 10 NIPs (Table 1; Fig. 1A). To understand the evolutionary dynamics of AQPs, with the Arabidopsis AQPs as query, we searched the genomes of 19 species representing all major lineages of the plant kingdom, including red algae and green plants. This identified a total of 524 AQPs, and a follow-up phylogenetic analysis clustered them into five major subfamilies, with 14 not belonging to any major subfamily. The TIP, PIP, and NIP subfamilies seemed to contain more members than the SIP and XIP. Next, we inferred the potential first ancestral node containing different AQP subfamilies along plant lineages following a pipeline previously reported (Fig. 1B) [36, 37]. We found that both SIPs and XIPs first appeared in chlorophytes, confirming the algal origin of these two subfamilies. However, the XIP subfamily was not detected in gymnosperms, monocots, and *A. thaliana*, indicating that after the origin, the subfamily genes might be frequently lost during the evolution of different plant lineages. Consistent with this, we observed nine genes without any obvious subfamily classification from early lineages (called `other' here: four from Rhodophytes, one from Chlorophytes, two from Charophytes, one from Bryophytes, and one from the basal angiosperm *Nymphaea colorata*). We also found five `orphan' genes from Chlorophytes (four) and Charophytes (one) clustering together with the PIP/TIP/NIP groups but not belonging to any of them. Agreeing with a previous notion [38], NIPs first appeared in charophytes and could be clustered into four groups in angiosperms. TIP subfamily diverged into three groups (TIP2, TIP4, and TIP1/TIP3) after the Lycophytes with the TIP1/TIP3 further divided into TIP1 and TIP3 groups in seed plants. Both TIPs and PIPs shared a common ancestor in chlorophytes and diverged in charophytes, while the PIPs further diverged into PIP1 and PIP2 groups from the Bryophytes lineage. In general, these suggest that the major AQPs are ancient proteins in early lineages and have diversified significantly from lycophytes on and especially in ferns and the seed plants (Fig. 1A and B).

**Table 1:** Species information and *AQP* subfamilies (*SIP*, *XIP*, *TIP*, *PIP*, and *NIP*) for the species used for phylogenetic clustering.

**Lineage**	**Species**	**Other**	**SIP**	**XIP**	**Orphan**	**PIP**	**TIP**	**NIP**	**Total**
Rhodophytes	*Porphyra umbilicalis*	4	0	0	0	0	0	0	4
Chlorophytes	*Chlamydomonas reinhardtii*	0	1	0	2	0	0	0	3
Chlorophytes	*Coccomyxa subellipsoidea*	1	1	1	2	0	0	0	5
Charophytes	*Chara braunii*	0	1	0	0	0	7	0	8
Charophytes	*Klebsormidium nitens*	1	1	0	1	9	3	2	17
Charophytes	*Mesostigma viride*	1	1	3	0	0	0	0	5
Liverworts	*Marchantia polymorph*	0	4	1	0	20	13	1	39
Mosses	*Physcomitrium patens*	1	2	2	0	10	6	6	27
Lycophytes	*Selaginella moellendorffii*	0	1	1	0	3	5	9	19
Ferns	*Ceratopteris richardii*	0	4	3	0	6	10	13	36
Gymnosperms	*Thuja plicata*	0	3	0	0	23	14	10	50
Gymnosperms	*Ginkgo biloba*	0	5	0	0	9	12	19	45
Angiosperms	*Amborella trichopoda*	0	4	0	0	5	7	7	23
Angiosperms	*Nymphaea colorata*	1	4	1	0	7	8	5	26
Angiosperms	*Zea mays*	0	10	0	0	14	18	12	54
Angiosperms	*Oryza sativa*	0	2	0	0	13	11	12	38
Angiosperms	*Solanum lycopersicum*	0	4	6	0	15	11	12	48
Angiosperms	*Arabidopsis thaliana*	0	3	0	0	14	11	10	38
Angiosperms Rosaceae	*Rosa wichuraiana* `Basye's Thornless'	0	4	3	0	10	10	12	39
	*Rosa chinensis* `Old Blush'	0	3	6	0	10	8	11	38
	*Rosa rugosa*	0	3	3	0	10	9	12	37
	*Fragaria vesca*	0	3	2	0	10	9	16	40
	*Malus × domestica*	0	5	2	0	15	13	14	49
	*Prunus armeniaca*	0	3	5	0	18	11	14	51
	*Pyrus ussuriensis × communis Zhongai*	0	5	4	0	11	9	13	42
	*Rubus occidentalis*	0	4	1	0	9	8	9	31
Total counts	26 species	9	81	44	5	241	213	219	812

### AQPs feature dynamic variation in protein domain structure along plant lineages

To further understand the evolutionary pattern of AQPs, we subsequently screened the conserved domains of all AQP subfamilies (Fig. 1B and Figs S1–S7). The Rhodophyte AQPs featured two conserved domains (5 and 6) that were shared with all other plant lineages. The SIP subfamily proteins gained a new domain (1) that was not detected in XIPs. However, the XIP subfamily proteins had evolved three additional domains (2, 4, 7) that were also present also in NIPs and TIPs and the ancestors leading to TIPs and PIPs. However, PIPs lost the domain 7. The NIP and TIP subfamilies shared the same conserved domain structure but likely have different origins, as NIPs possibly originated from a single horizontal gene transfer (HGT) event from bacteria to plants [39], while the TIPs could be traced back to chlorophytes with PIPs. Notably, the PIP subfamily had two unique and highly conserved domains (3 and 9), indicating that the PIP subfamily may have evolved new functions after the appearance of lineage Charophytes.

Taking these together, AQPs appear already in early plant lineages but have diversified strongly after the transition to land growth. Next, we investigated the dynamic evolutionary pattern at the family level, taking the biologically and economically important Rosaceae as one example.

### Rosaceae AQPs are highly conserved in protein sequence but can vary in copy number

**Figure 2:**
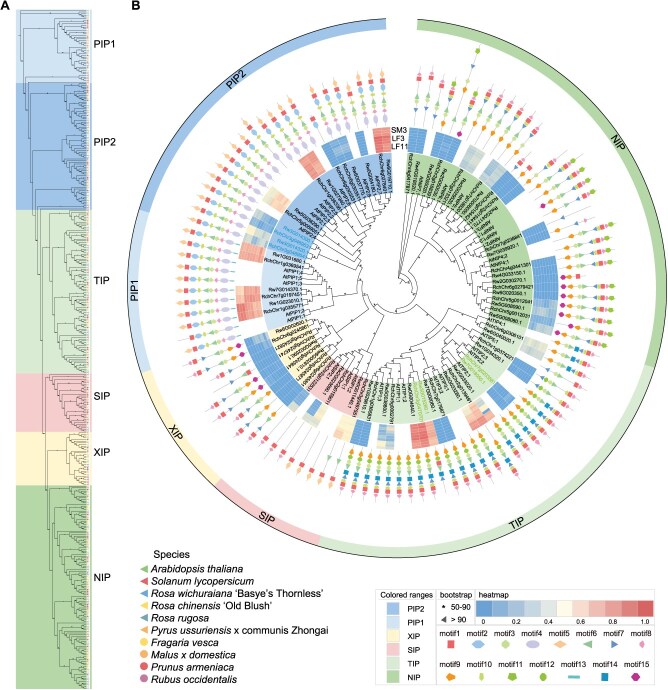
Rosaceae AQPs are relatively conserved in protein sequences and subfamily structure. (A) Phylogenetic classification of Rosaceae AQPs into five subfamilies, with PIP subfamily further divided into PIP1 and PIP2 groups. An unrooted maximum likelihood phylogenetic tree is shown with the subfamilies (namely PIP1, PIP2, TIP, SIP, XIP, and NIP) displayed on the right, distinguished by different colors, and the name of each subfamily labeled. The * and triangle indicate bootstrap supports of 50%–90% and >90%, respectively. Shapes represent the species (the same for B). (B) Circular illustration of protein features of AQPs in *Rosa chinensis* `Old Blush' (OB/Rch) and *R. wichuraiana* `Basye's Thornless' (BT/Rw). From inside to outside, subfamilies, protein domain structure, an expression heatmap in SM3 of March (SM3) and leaves (LF3 and LF11 for samples collected in March and November, respectively), and the unrooted phylogeny. Fifteen motifs are represented by shape patterns in different colors, respectively.

A total of 325 *AQP* members were identified in eight Rosaceae species, phylogenetically classified into five subfamilies, including PIP, TIP, NIP, SIP, and XIP (Fig. 2A). The AQP numbers varied among species, with *R. wichuraiana* (BT; 37), *R. chinensis* (OB; 38), *Rosa rugosa* (37), *Fragaria vesca* (40), *Malus × domestica* (49), *Prunus armeniaca* (51), *Pyrus ussuriensis × communis* Zhongai (42), and *Rubus occidentalis* (31) (Table 1). *Rubus occidentalis* harbored the fewest AQPs (31), while *P. armeniaca* contained the most (51). *Prunus armeniaca* had the highest number of PIPs (18), doubling that of *R. occidentalis*. *Malus × domestica* possessed 13 TIPs, 1.6-fold more than *R. occidentalis*, whereas *F. vesca* had the maximum number of NIPs (16), ~1.8-fold higher than *R. occidentalis*. The SIP subfamily showed minimal variation, with *R. wichuraiana* containing the fewest SIPs (2). In contrast but consistent with the pattern in major lineages of plants (Table 1; Fig. 1), the XIP subfamily exhibited considerable variation, with *R. chinensis* harboring six XIPs and *R. occidentalis* only one. These findings hence highlight significant interspecific variation of the AQPs in Rosaceae.

To explore the diversity in *AQP* expression and gene structure, we conducted a detailed comparison of AQPs for OB and BT with Arabidopsis as reference. Again, the AQPs in both species were classified into the five major subfamilies with PIP further divided into PIP1 and PIP2 subgroups (Fig. 2B). A MEME analysis identified 15 conserved motifs and most of them were conserved within subfamilies, while only motif 1 presented ubiquitously in most AQPs except one RwNIP (Rw7G039920.1) and two RcNIPs (RchChr7g0238841 and RchChr2g0168391). Additionally, these AQPs could vary in protein length, molecular weight, and isoelectric point (Tables S1 and S2). Phosphorylation site prediction indicated that, except one XIP and one NIP, all RcAQPs and RwAQPs contained Ser, Thr, and Tyr phosphorylation sites. Most AQPs were predicted to be plasma membrane-localized, with some localized to vacuoles, chloroplasts, and cytoplasm.

In summary, there is a high conservation in protein features within AQP subfamilies, a pattern that drives us to consider whether some subfamilies are under directional selection.

### Rosaceae *PIP2* and *SIP* subfamilies have experienced strong positive selection

**Figure 3:**
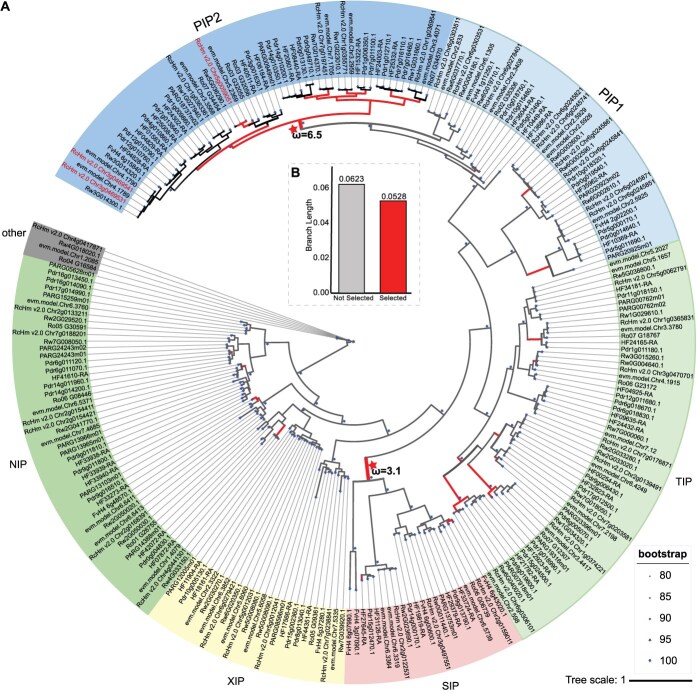
Rosaceae PIP2 and SIP subfamilies have experienced strong positive selection. (A) An unrooted maximum likelihood phylogeny of Rosaceae AQPs highlights positively selected branches, which were shown in bold and colored with different colors (FDR correction; *q* < 0.05). Bootstrap values (shown as blue stars on nodes, with sizes indicating confidence levels) are provided. The scale bar indicates substitutions per site. The ω value refers to the ratio of nonsynonymous substitutions (*dN*) to synonymous substitutions (*dS*). The ω values of PIP2 and SIP subfamilies are marked on the branches. (B) The insert graph marked the mean branch lengths (substitution/site) for selected (right boxplot) and nonselected (left boxplot) branches.

The directional evolution of 248 Rosaceae AQPs were tested for the ratio (ω) of nonsynonymous substitutions (*dN*) against the synonymous substitutions (*dS*; Fig. 3A). Among all branches, 28 branches (11.3%) were identified as statistically significant and under positive selection [corrected false discovery rate (FDR), *P*-value <0.05], while the remaining 220 branches (88.7%) showed no significant evidence of positive selection. Mean branch length of positively selected branches was 0.0528, which was comparable to that of nonselected branches (0.0623), suggesting that positive selection was not exclusively associated with faster evolving lineages (Fig. 3B). Most positively selected branches were distributed randomly along deep branches. Surprisingly, two subfamilies, i.e. the PIP2 and the SIP, were under strong positive selection with ω being 6.5 and 3.1, respectively, indicating that these two subfamilies might have evolved directional molecular and/or cellular function. Specifically, the PIP2 subfamily shows a higher frequency ratio of nonsynonymous to synonymous substitutions (*dN/dS* ratio), suggesting that genes within this lineage have undergone positive selection. This adaptive pressure may reflect shifts in function or environmental adaptation, emphasizing the PIP2 subfamily's evolutionary trajectory.

### Rosaceae *AQP*s feature a good synteny but variable intron–exon structure across subfamilies

To reveal the evolutionary dynamics of Rosaceae *AQP*s, we examined the gene collinearity pattern using the *PIP2*s within a prickle-related QTL1 as examples [35]. In this QTL1 vicinity, we additionally identified one *TIP2* gene (*RchChr3g0470701/Rw3G015260*) ~95 genes downstream of the tandemly duplicated *PIP2*s (Fig. S8A). Both *TIP2* and *PIP2A/B* genes featured a high level of synteny and were located on the same chromosome of the four species in Rosoidae (*Rubus idaeus*, *F. vesca*, OB, and BT). However, Maleae plants (*Pyrus pyrifolia*, *M. domestica*, and *Crataegus pinnatifida*) in Amygdaloideae seemed to have duplicated their *PIP2*s via an ancient whole-genome duplication (WGD) event, with one *PIP2* collocated with the *TIP2* orthologs on one chromosome [40, 41], while the duplicated *PIP2*s sit on a separate chromosome. *Prunus campanulata* had its *TIP2* and one *PIP2* on chromosomes 06 and 07, respectively. Interestingly, *Dryas drummondii*, the sister to the rest of the Rosaceae plants, had only one *PIP2* and one *TIP2* on the same scaffold 270 with very good synteny to both Rosoideae and Maleae plants. However, the two tandemly duplicated *PIP2*s and the *TIP2* were respectively mapped to chromosomes 3 and 12 of the outgroup *Ziziphus mauritiana*, indicating that the genomic region harboring the *PIP2*s and *TIP2* had experienced strong genomic reorganization in some Rosaceae plants.

To elaborate in detail on the evolutionary pattern of the *AQP*s in Rosaceae, we performed a genome-wide chromosomal painting to identify their distribution pattern on chromosomes of two *Rosa* species, OB and BT. This analysis revealed nonuniform distribution of the 37 *RwAQP*s, with chromosome 2 harboring the highest gene density (eight loci) and chromosome 4 the lowest (two loci) (Fig. S8B). Three *RwAQP*s remained unmapped to the seven chromosomes. *RcAQP*s and *RwAQP*s featured a good synteny along chromosomes of BT and OB (Fig. S9A). Comparative synteny analysis identified 35 orthologous pairs between *RwAQP*s and *RcAQP*s, with three *RcAQP*s (*RchChr2g0139491*, *RchChr2g0168391*, and *RchChr5g0012041*) exhibiting tandem duplication events relative to their *RwAQP* counterparts. Corroborating with the high similarity among protein structures (Fig. 2B), purifying selection seems to be dominating the evolution of AQPs, especially these PIP and TIP subfamilies, as evidenced by the ratios of nonsynonymous versus synonymous changes (*dN/dS*) always <1 across all orthologs (Fig. S9B; Table S3).

**Figure 4:**
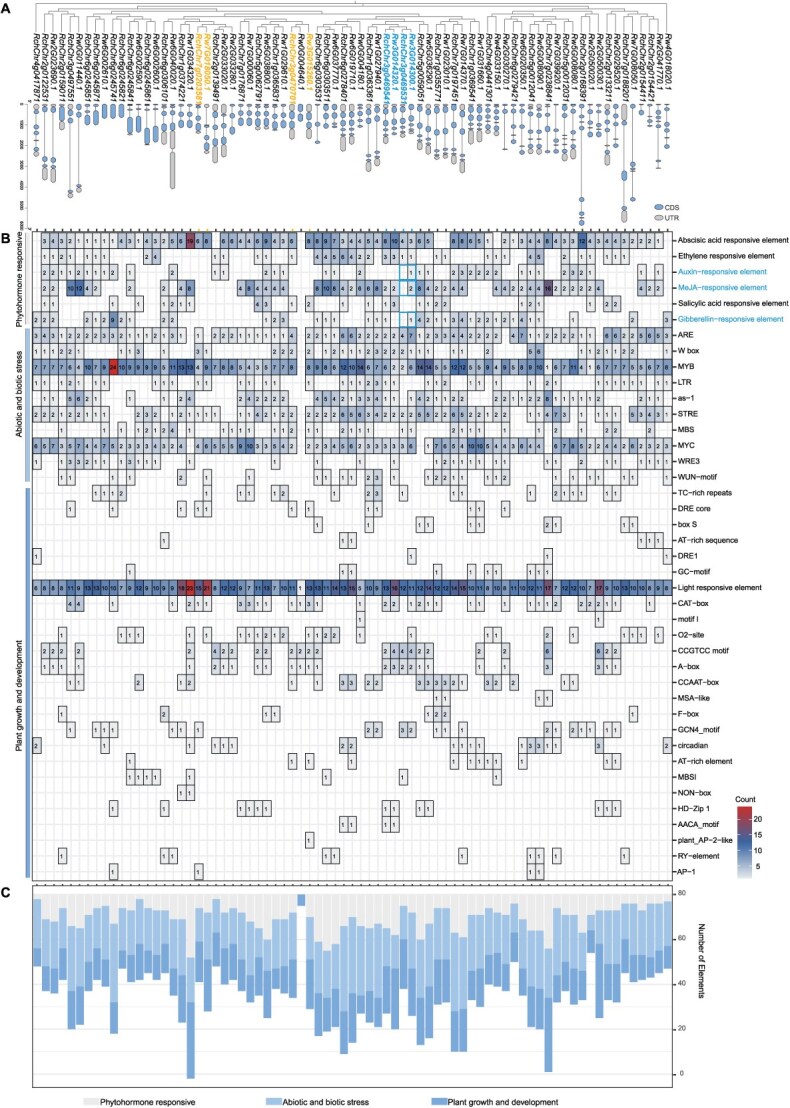
Structural conservation and regulatory divergence of *AQP* pairs in BT and OB. (A) An unrooted maximum likelihood phylogeny of *AQPs* in OB and BT, incorporating the gene structure feature. Arrowed lines denote the orientation and the gene architecture. Coding sequences (CDS) and untranslated regions (UTR) are represented by ellipses of different colors. Two *PIP2* pairs and *TIP* pairs located in the QTL intervals of chromosome 3 and chromosome 7 are shown in bold blue and bold orange, respectively. (B) The heatmap of the count distribution of *cis*-regulatory elements in the 2-kb promoter regions of *AQPs*. Elements are categorized into three classes, phytohormone-responsive, abiotic and biotic stress responsive, and plant growth and development. The number of each element is marked in the box and indicated by a color gradient. **C**. The stacked bar plots representing the proportion of each class of element. From top to bottom, the bars represent phytohormone responsive elements, abiotic and biotic stress-responsive elements, and plant growth and development related elements.

A follow-up gene structure analysis uncovered substantial intron/exon variations across *AQP* subfamilies, with transcript lengths ranging from 708 to 5856 bp (Fig. 4A). While intron numbers varied markedly (0–6), there are obvious subfamily-specific patterns. All *PIP*s have predominantly three introns, except four introns in *Rw0G004180* in BT and *RchChr6g0303511* and *RchChr6g0303531* in OB. Most of the *TIP*s, *SIP*s, and *XIP*s feature two introns. However, *RwNIP*s frequently have four introns, while *RcNIP*s are highly variable with one to seven introns.

Together, despite the moderate variation in intron–exon numbers, subfamily-level *AQP*s are highly conserved in Rosaceae plants. This drives us to identify the sequence changes in noncoding regions, especially the upstream promoters.

### Tissue-specific expression corroborated with prevalent *cis*-motif variation within promoters of rose *AQP*s

A systematic analysis of *AQP* expression identified a strong variation in different tissues of Arabidopsis with 14 genes specific for root, 5 for flower, 3 for second internode of stem, 4 for the dry seed, and 2 for shoot apex (Fig. S10). A follow-up analysis using single-cell transcriptome of Arabidopsis roots further revealed a strong specific expression in root cortex cells for *PIP1;3*, *PIP2;2*, and *TIP4;1* (Fig. S11). And the same pattern was observed for roses. There is frequent expression divergence between the syntenic *AQP* pairs in the shoot apical meristems of March (SM3) and leaf materials in March (LF3) and November (LF11) of OB and BT plants, indicating that promoter sequences may differ between species (Fig. 2B). Indeed, a systematic profiling of 2-kb promoter regions revealed distinct *cis*-element compositions, with a notable divergence in hormone-responsive motifs, between syntenic *RwAQP* and *RcAQP* pairs (Fig. 4B). The light-responsive elements were universally present (411 events for *RwAQP*s and 412 for *RcAQP*s), while stress- and development-related elements exhibited species-specific enrichment patterns.

Previously we have identified two *PIP2*s (*Rw3G014300/RchChr3g0469531* for *PIP2A* and *Rw3G014320/RchChr3g0469541* for *PIP2B*) featuring significant expression variation within the prickle-related QTL1, in which an additional *AQP*, *Rw3G015260*/*RchChr3g0470701*, was identified to be a member of the TIP subfamily (*TIP3*s; Figs 2B and 4A) [35]. *Rw3G014300* contained one auxin-, two methyl jasmonate-(MeJA), and one gibberellin-responsive element in its promoter, while these motifs were entirely absent in its ortholog *RchChr3g0469531*. On the other hand, a gibberellin-responsive element was uniquely present in the promoter of *RchChr3g0469541* but not *Rw3G014320*. *RchChr3g0470701* harbored one ethylene responsive element, which was absent in the *Rw3G015260* promoter. Another *AQP* pair (*Rw7G008050/Rch7g0202581*; *TIP2*s) in the second QTL also featured significant variation in numbers of *cis* elements related to biotic and abiotic stresses (Fig. 4C). In general, these results suggest that rose *AQP*s may interact differentially with hormones in their expression, a pattern known for *AQP*s in both *Arabidopsis* and rose [19–25].

### Species-specific spatial–temporal expression for *AQP*s during prickle development

**Figure 5:**
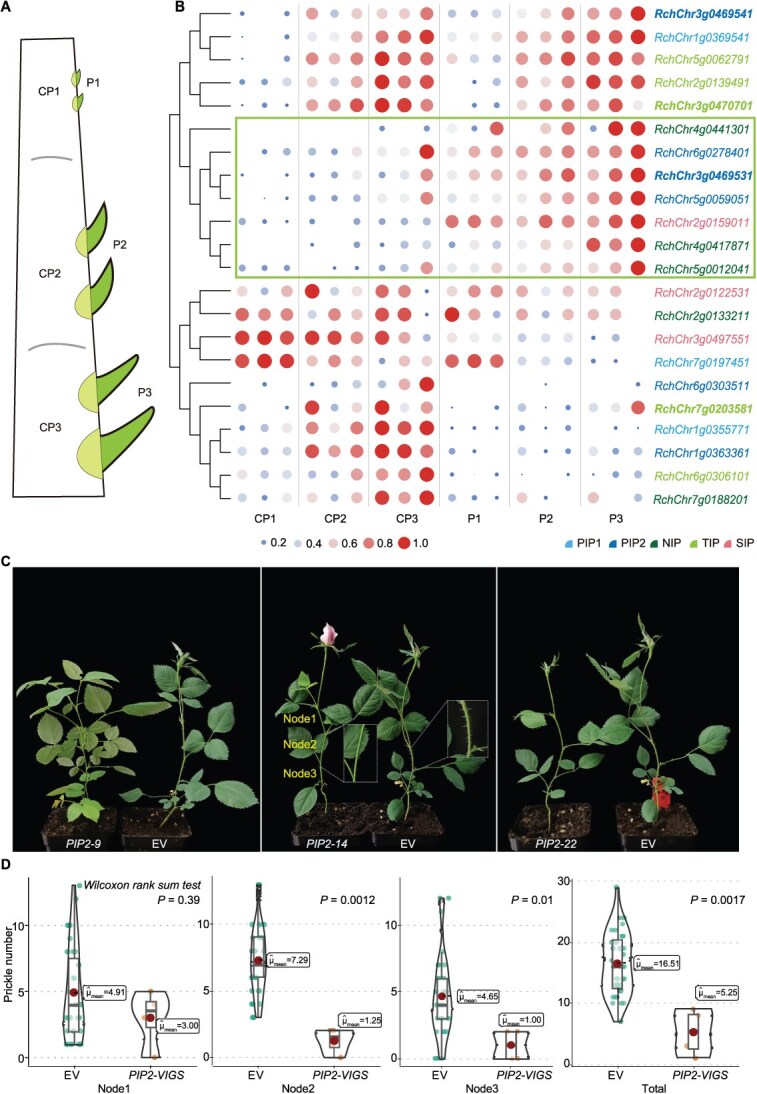
Tissue-specific expression patterns of *AQP*s reveals a critical role for *PIP2* in the development of rose stem prickles. (A) RNA-seq sampling schema for two types of tissues (P for prickles and CP for cortical tissues under prickles) at three developmental stages in rose (P1 to P3 and CP1 to CP3 on different internodes). (B) Heatmap displaying the spatiotemporal expression profiles of *AQP*s with colors and circle size representing expression levels ranging from low to high. The four *AQP*s located within the QTL1 region are highlighted in bold names. The seven genes showing prickle-specific expression are highlighted with a box. (C) Representative images of *R.* `Samantha' silenced with anti-*PIP2* probes (*PIP2–9*, *PIP2–14*, and *PIP2–22*; left) or empty vectors (EV; right). Inserts for line *PIP2–14* show the enlarged photos of node 2, counted from the pedicels. The *R.* `Samantha' plants had developed at least three new internodes, with the majority having formed flower buds. (D) Violin plots showing the prickle numbers on Node 1, 2, 3, and three nodes together (total) of roses silenced with EV and anti-*PIP2* (*PIP2-VIGS*). *P-*values tested with Wilcoxon rank-sum test are given.

To delineate the functional roles of AQPs in prickle morphogenesis, we explored a tissue-specific transcriptome profiling that was generated by profiling the gene expression across three developmental stages of prickles (P1 for early, P2, and P3 for late stages) and their underlying cortex tissues (CP1 for early, CP2, and CP3 for late) in a prickle-rich species, *Rosa gigantea* (Fig. 5A). In this profile, expression for 22 *AQP*s (9 *PIP*s, 5 *TIP*s, 5 *NIP*s, and 3 *SIP*s) was detected in three different patterns (Fig. 5B). Two *PIP*s (*RchChr3g0469541* and *RchChr1g0369541*) and three *TIP*s (*RchChr5g0062791*, *RchChr2g0139491*, *RchChr3g0470701*) increased their expression following developmental stages but without difference between P and CP tissues. Seven *AQP*s, including *RchChr3g0469531*, a PIP2 subfamily member that locates within the QTL1 region, showed specific expression in prickles, and three of them (*RchChr3g0469531*, *RchChr6g0278401*, and *RchChr5g0059051*) featured an increased expression following prickle maturation. The remaining 10 *AQP*s had a predominant expression in CPs with the expression for *RchChr3g0497551* decreased and the expression for *RchChr1g0355771*, *RchChr1g0363361*, *RchChr6g0306101*, and *RchChr7g0188201* increased following CP development, respectively.

Interestingly, the *AQP* gene pairs at subfamily level also showed a tissue-specific expression pattern between SM3 and leaves at different seasons (LF3 and LF11) for OB and BT (Fig. 2B) [35]. While most orthologous pairs exhibited conserved expression, notable exceptions included in the *PIP* subfamily a conserved leaf expression in *Rw3G014320/RchChr3g0469541* pair but with BT ortholog gaining SM3 expression, in the *TIP* subfamily a 2.1-fold SM3-enrichment in *Rw5G03880* (BT) versus OB counterpart *RchChr5g0062791*, in the *NIP* subfamilies a reciprocal organ specificity (*Rw5G0080901* in leaf against *RchChr5g0012041* in SM3), and in the *SIP* subfamily with a conserved SM3 expression except *RchChr2g0159011* having an extra expression in leaf.

In summary, rose *AQP*s feature a highly diverged spatial–temporal expression like Arabidopsis. This dichotomy between orthologs aligns with *cis-*regulatory element variations, suggesting evolutionary rewiring of transcriptional networks governing *AQP* spatial regulation.

### Rose *PIP2* plays essential roles in prickle development

The prickle-specific expression of *RchChr3g0469531* following maturation prompted us to ask whether it played a role in regulation of the prickle morphogenesis (Figs 2 and 4) [35]. For this, we performed a VIGS experiment in the prickly *R*. `Samantha', one variety frequently used for functional tests [42, 43]. Total RNA was extracted from the leaves of newly formed second internode of the plants, followed by reverse transcription and Quantitative Real-time Polymerase Chain Reaction (qPCR) analysis to determine the expression level of *RcPIP2A*. The expression of *RchChr3g0469531* was remarkably reduced to ~25% level (two-tailed Student's *t*-test, *P* < 0.0001; Fig. S12). Compared to the `Samantha' plants infected with empty vector (EV), all the VIGS plants featured a reduced number of prickles on the nodes 2 (Wilcoxon rank sum test, *P* = 0.0012) and 3 (*P* = 0.01) below the pedicels (Fig. 5D). There was no significant difference on the node 1 (*P =* .39), which was just below the pedicel, indicating that the silencing effect might become weak over time after infection. Under our growth conditions, no other obvious phenotypical variation was observed, suggesting that, corroborating with its specific expression in prickles, *RchChr3g0469531* plays pivotal roles in the regulation of roses.

### A hypothetical model of PIP2-mediated cell expansion driving prickle development

**Figure 6:**
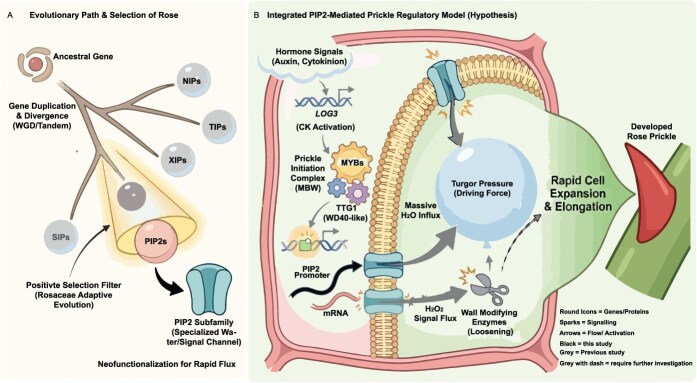
Evolutionary origin and proposed PIP2-mediated regulatory model of rose prickles (Solid black arrows indicate activation or transport; `P' denotes phosphorylation; PM, plasma membrane). (A) A proposed evolutionary path for prickle formation genes in *Rosa*. Ancestral genes underwent duplication events, giving rise to diversification of aquaporin subfamilies. Some duplicated genes (such as SIPs and PIP2s) were subsequently shaped by positive selection during Rosaceae adaptive evolution, leading to neofunctionalization. (B) A hypothetical working model of PIP2-mediated cell expansion driving prickle development. Hormonal signals (Auxin, Cytokinin) activate downstream components, including *LOG3* (involved in cytokinin activation), MYB transcription factors, and the WD40-repeat protein TTG1. This transcriptional complex likely binds the *PIP2* promoter, upregulating *PIP2* subfamily gene expression. The resulting PIP2 proteins function as specialized water/signal channels facilitating massive H₂O influx. Concurrently, wall-modifying enzymes loosen the cell wall, while H₂O₂ flux and hormone signals further amplify the response. The resultant turgor pressure provides the driving force for rapid cell expansion and elongation, ultimately leading to prickle protrusion.

To investigate the molecular origin and regulatory mechanism underlying prickle morphogenesis in Rosa, we constructed an integrated evolutionary and mechanistic model. As depicted in Fig. 6A, we propose that the genes arose from ancestral sequences through genomic duplication events, including WGD and tandem duplication. These events led to the diversification of AQP subfamilies. The subsequent 2 subfamilies (PIP2 and SIPs) undergone the positive selection during the adaptive evolution of the Rosaceae family, likely driving the neofunctionalization of these paralogs.

Building on this evolutionary framework, we propose a hypothetical working model for PIP2-mediated prickle development (Fig. 6B). Our model posits that developmental cues, specifically auxin and cytokinin signals, activate a transcriptional regulatory complex. This involves *LOG3*, which mediates cytokinin activation [33, 44], and the synergistic action of MYB transcription factors [24, 45] and the WD40-repeat protein TTG1 [35]. We hypothesize that this complex binds the PIP2 promoter, driving the expression of *PIP2* subfamily genes. The synthesized *PIP2* proteins are then proposed to function as specialized water (and potentially signal) channels at the plasma membrane [46]. Their activation facilitates a massive influx of water, generating substantial turgor pressure. This driving force, combined with the activity of cell wall modifying enzymes that loosen the wall structure, and the modulating effects of H₂O₂ signal flux, culminates in rapid cell expansion and elongation, ultimately leading to the physical protrusion of the prickle [47]. This model is built upon three core scientific hypotheses. Transcriptional Coupling Hypothesis: Key transcription factors (the MYB complex) governing lateral organ development control not only cell identity but also physical growth dynamics. MYB serves as the direct upstream `switch' for *PIP2* genes, ensuring that hydraulic growth is synchronized with developmental programming [46]. Turgor-driven Outgrowth Hypothesis: The initiation and elongation of prickles involve extremely rapid cell expansion. We hypothesize that *PIP2* is the rate-limiting step for water permeability at these specific sites, and its expression level directly determines the final morphogenesis of the organs [25]. Signaling Homeostasis Hypothesis: PIP2 facilitates the transport of both water and H_2_O_2_ [47]. We hypothesize that lateral organ development depends on a specific intensity of redox signaling, and the stability of the PIP2 protein (regulated by factors like CPK28) is essential for maintaining this signaling balance [48].

## Discussion

Morphological innovation can be created by changes in protein sequences and/or structure, by expression alteration either in amount or in place via sequence mutation in regulatory regions, e.g. the promoters, or by rewiring preexisting protein partners and related gene regulatory networks. Here, we reveal the evolutionary pattern of *AQP*s with the first ancient appearance in red algae (Rhodophyte) followed by strong diversification in green plants including ferns and seed plants. Taking the Rosaceae as an example, we demonstrate that the AQPs are highly conserved in protein structure but display significant regulatory divergence, particularly in *cis*-element composition and spatiotemporal expression patterns, which may underlie their roles in prickle development, thereby serving as a target of future breeding [49].

### Ancient but dynamic evolution of AQPs in major plant lineages

AQPs have been considered as ancient water channel proteins present in all lineages of life organisms with essential roles [50]. With 28 species featuring high-quality genomes as representatives across all major plantae lineages, we showed that the first appearance of *AQP*s was in red algae and the diversification of *AQP*s into five major subfamilies could date back to charophytes, with *XIP*s being frequently absent in gymnosperms, monocots, and some dicots like *Arabidopsis* (Table 1; Fig. 1) [38]. The absence of *NIP* subfamily in green algae and the parallel to other subfamilies agrees with a hypothesis that these *NIP*s might have originated from an HGT event from bacteria [38, 39]. Notably, the copy number of the *NIP*, *TIP*, and *PIP* subfamilies significantly increased in seed plants except the basal angiosperms like *Amborella trichopoda* and *N. colorata*, a pattern that seems to be unlikely caused by the WGD events that occurred in both gymnosperm and angiosperm (Table 1; Fig. 1). Rather, this may be due to the environmental shifts causing most angiosperm lineages to experience independent WGD events during the time between the Cretaceous and Paleogene stages [51]. Furthermore, our protein structure analysis reveals that the AQPs contain two very ancient domains (5 and 6) in Rhodophytes and later increased their conserved domains to at least three for SIPs in Chlorophytes and a maximum of nine for PIPs in Charophytes across the evolution of plants (Fig. 1 and Figs S1–S7). In addition, PIPs have evolved two subfamily-specific domains (3 and 9), suggesting that these proteins might have diverged functions [38, 52, 53]. The separation of the PIP subfamily into two groups (PIP1 and PIP2) likely occurs after the appearance of Charophytes and these groups feature high protein sequence conservation (Fig. 1 and Figs S6 and S7). Both groups harbor the PIP-specific domains 3 and 9, suggesting a vital role in their function [54]. In general, AQP evolution is rather ancient but dynamic in broader lineage level of the plant kingdom, a pattern also seen at the family level in angiosperms.

### Conservation of protein coding regions and divergence of regulatory sequences for Rosaceae *AQP*s

The identification of 325 AQPs across eight Rosaceae species highlights the evolutionary conservation of this gene family, consistent with their critical roles in water and solute transport in both plants and animals [1, 55–57]. While protein sequences and phosphorylation motifs are largely conserved, interspecific variation in gene copy numbers such as the expansion of *PIP*s in *P. armeniaca* and *NIP*s in strawberry suggests lineage-specific evolution may happen due to varying environmental niches (Table 1; Fig. 2). The dominance of purifying selection (*dN/dS* < 1) further underscores the functional constraints on AQP evolution (Fig. 3 and Fig. S9). It should be noted that the PIP2 and SIP subfamilies were under strong positive selection (Fig. 3), though no clue on why or how they were selected presents.

However, a sequence comparison followed by *cis*-element screening in promoters revealed substantial divergence in hormone-responsive motifs (e.g. auxin, gibberellin, and jasmonate) between syntenic AQP pairs in *R. chinensis* (OB) and *R*. *wichuraiana* (BT) (Fig. 4B and C), aligning well with known results in both *Arabidopsis* and roses, in which hormonal regulation of *AQP*s modulates organ-specific growth and stress responses [19–26]. Our findings here extend this paradigm to prickle development as seen by the species-specific *AQP* expression between prickle and the underlying cortical tissues (Figs 2, 4, and  5). And indeed, agreeing with this, some Arabidopsis *AQP*s are specifically expressed in cortex cells of roots (Figs S10 and S11) [58, 59].

Similarly, *cis*-element divergences between OB and BT *AQP* promoters may underscore their integration into hormonal and stress-responsive GRNs. For example, the absence of auxin- and jasmonate-responsive motifs in *RchChr3g0469531* of OB versus its ortholog in BT may reflect an evolutionary rewiring of *AQP* expression to balance growth–defense trade-offs (Fig. 4B and C). Such regulatory plasticity aligns with studies showing AQP interactions with phytohormones like ethylene and ABA to modulate stomatal closure and drought responses [11, 25, 48]. In roses, ethylene-responsive *RhPIP1;1-RhPIP2;1* interaction regulates petal morphogenesis [26], suggesting that a conserved mechanism can be retuned for prickle development.

### *AQP* expression dynamics coordinate regulation of prickle development and water homeostasis

Prickles, frequently misconsidered as modified trichomes, serve dual roles in defense and water storage during early development [2]. Our transcriptome profiling in *R. gigantea* revealed stage- and tissue-specific *AQP* expression patterns, with *PIP2*s (e.g. *RchChr3g0469531*) showing prickle-enriched upregulation during maturation. This parallels the role of *PIP2* homologs in other systems, such as *ZmPIP2;5*, which affects both the water sensitivity and leaf development under certain growth conditions [60–62]. Intriguingly, the high relative water content in developing prickles (84%) compared to cortical tissues (76%) further supports their role as transient water reservoirs [35], a feature potentially regulated by PIP2-mediated water flux. Further functional validation of *RchChr3g0469531* via the VIGS approach in rose `Samantha' demonstrated its prickle-specific regulatory role, with its silencing significantly reducing prickle density at early developmental stages (Fig. 5C). This mirrors the findings in *Arabidopsis*, where AQPs coordinate cell expansion through vacuolar dynamics [18], and highlights the evolutionary rewiring of AQP functions in nonmodel species. In line with these, litchi *MYB306* delays postharvest water loss and browning by repressing *LcPIP2;4* expression [45]. Several transcriptome profilings have identified the coexpression of transcription factors like *MYB, NAC, MADS-box* with prickle development [34, 63, 64], but whether these transcription factors interact with the PIP2s needs to be identified.

However, key questions remain. While our VIGS assay implicates *PIP2;1* in prickle development (Fig. 5) [35], whether this role is directionally selected (Fig. 3) and the mechanistic links between *AQP*s and prickle ontogeny, particularly in the contexts of hormonal regulation and transcription factors, seem to be rather complex. In *Zanthoxylum armatum* multiple hormones including cytokinin, auxin, jasmonic acid, and gibberellins participate in the regulation of prickle initiation and/or development at least partially via interacting with transcription factor NAC93 [65, 66]. Auxin can promote lateral root emergence by regulating the spatial and temporal expression of *PIP2;1* [19]. Exogenous MeJA treatments increase root hydraulic conductivity, a process accompanied by an enhanced phosphorylation state of PIP2 in bean, tomato, and *Arabidopsis* [67]. Drought treatment of poplar roots altered the levels of abscisic acid and cytokinin along with *PIP1* and *PIP2* expression [68]. In roses, *PIP2* interacts with ethylene in regulating water transport and cellular turgor of petal [25, 26]. These studies highlight the close interactions between *PIP2s* and hormonal signaling in modulating cellular water transport and therefore the regulation of a range of developmental and adaptive responses [69]. Further investigation of its interactions with broader regulatory networks like the one in flowering time regulation should provide novel insights into the *PIP2* function in plant development and adaptation [70].

A recent study identified the involvement of *LONELY GUY 3* (*LOG3; Rw3G014450*/*RchChr3g0469741*), a key cytokinin biosynthesis gene, which colocalizes with *Rw3G014300* in BT and *RchChr3g0469531* in OB within the prickle-density QTL1 region [33, 35]. Future investigations could explore whether *PIP2;1* and *LOG3* can interact to fine-tune prickle density in roses. Notably, convergent mutations in *LOG3* have been repeatedly associated with prickle-free phenotypes across diverse species, including eggplant, barley, jujube, and roses [33]. It would be interesting to test the idea of whether LOG3s, like PIP2s, are also directionally selected in these species, since positive selections often link tightly with morphological innovations [71, 72]. Another parallel but compelling question is how has the sequence variation in the promoters of *Rw3G014300/RchChr3g0469531* evolved among different *Rosa* species and other prickly angiosperms? Additionally, the development of CRISPR-edited *AQP* variants that significantly reduce prickle density without compromising water-use efficiency, akin to the *cop1–21* point mutation in *Arabidopsis*, which causes early flowering without growth penalties [73], represents a promising avenue for breeding novel rose germplasms. Such advances could facilitate industrial applications of roses and potentially other spiking crops, offering a dual benefit of improved horticultural traits and enhanced resource-use efficiency.

## Materials and methods

### Plant materials and growth conditions

BT and OB plants were maintained under natural light conditions in a greenhouse located in Kunming, Yunnan, China. *Rosa hybrida* ‘Samantha’ plantlets were propagated via tissue culture using nodal stem segments as explants following procedures previously reported [74]. Briefly, the explants from shoot apical stems with one node were cultivated for budding on Murashige and Skoog (MS) basic medium (Phyto Technology Laboratories, USA) supplemented with 30 g/l sucrose, 1.0 mg/l 6-benzylaminopurine, and 0.05 mg/l α-naphthaleneacetic acid (NAA) under controlled conditions (22 ± 1°C, 16-h light/8-h dark photoperiod) for 30 days under a 16-h/8-h light/dark photoperiod. Subsequently, shoots were transferred to half-strength MS medium containing 30 g/l sucrose and 0.1 mg/l NAA for root induction over an additional 30-day period. Rooted plantlets were acclimatized in pots containing a 1:1 (v/v) peat moss: perlite substrate under the same temperature and photoperiod conditions, with relative humidity maintained at ~60%.

### Genomic DNA extraction and total RNA extraction

Genomic DNA was isolated from the young leaves of OB and BT utilizing the Plant Genomic DNA Kit (CWBIO, China). Total RNA was extracted from juvenile prickles of OB using OminiPlant RNA Kit (DNase I; CWBIO, China). The RNA integrity was verified using gel electrophoresis on 1.0% nondenaturing agarose gels.

### Genome-wide identification and phylogenetic analyses of *AQP*s across major lineages of plant kingdom

AQP proteins from *A. thaliana* (The Arabidopsis Information Resource, TAIR) and *Solanum lycopersicum* (https://solgenomics.sgn.cornell.edu/organism/Solanum_lycopersicum/) were used as reference for homologous sequence identification (Basic Local Alignment Search Tool for proteins, BLASTp) from 18 species representing all major lineages of the plant kingdom (Table 1). Protein sequences were aligned against the reference database using DIAMOND (v2.1.10.164) with an E-value cut-off of 1e-5 [75]. Candidate sequences were further validated through Conserved Domain Database (CDD) screening for AQP domains (PF00230) [76]. Multiple sequence alignment was performed using MAFFT (v7.526) with automated parameter optimization [77]. Phylogenetic analysis was conducted using IQ-TREE (v2.3.6) (Efficient phylogenomic software by maximum likelihood) under the JTT + GAMMA model with 1000 bootstrap replicates [78]. Resultant trees were visualized using iTOL (v6.7.6) [79]. Conserved motifs were predicted using MEME Suite (http://meme-suite.org/) with a maximum of 10 motifs under default parameters.

### Genome-wide identification and phylogenetic and structural analysis of AQP genes in eight Rosaceae genomes

Genomic data for eight Rosaceae species (OB, BT, *R. rugosa*, *F. vesca*, *M. domestica*, *P. armeniaca*, *P. ussuriensis × communis* `Zhongai', and *R. occidentalis*) were retrieved from the Genome Database for Rosaceae (GDR) and PlantGIR [80, 81]. AQP homologs were identified through reciprocal BLAST searches TBtools (v2.152) and HMMER profiling (Pfam PF00230) (http://pfam.xfam.org/) [82]. Candidate sequences underwent additional validation via SMART and CDD analyses. Physicochemical properties, phosphorylation sites, and subcellular localization predictions were predicted using ExPASy [83], NetPhos 3.1 [84], and WoLF PSORT (https://psort.hgc.jp/), respectively.

Protein sequences were aligned using the MUSCLE module in MEGA7 [85], with phylogenetic trees constructed via maximum likelihood method in IQ-TREE with 1000 replicates of bootstraps. The MEME Suite was used to identify conserved motifs with a maximum number of 15 motifs.

Chromosomal localization maps were generated using the Gene Structure Display Server (v2.0) and TBtools [82, 86]. Collinearity analysis employed the MCscan module in JCVI with stringent parameters (e-value <1e-20, max_gaps = 15, min_collinear_blocks = 8) [87]. The sequences of orthologs gene pairs between OB and BT were first aligned with MUSCLE and the pairwise *dN/dS* ratios were calculated using the codeml in phasePAML (https://github.com/janinamass/phasePAML) [88].

### Positive selection analysis in AQPs

*AQP* gene sequences were retrieved from our genomic database. Multiple sequence alignment was performed using PRANK (v.170427). The codon-aware alignment option was employed to maintain reading frame integrity and improve alignment accuracy for subsequent evolutionary analyses. Following alignment, sequences underwent comprehensive quality control procedures. We developed custom Python scripts to perform the following processing steps: (i) all sequences were verified to maintain proper reading frames throughout the alignment. (ii) Internal stop codons were identified and removed using a sliding window approach across all sequences. Sequences containing >2% internal stop codons were excluded from further analysis. (iii) Columns containing >50% gaps were removed to reduce alignment noise while preserving phylogenetic information.

Maximum likelihood phylogenetic trees were constructed using IQ-TREE (v2.1.2) with the following parameters: -m TEST -bb 1000 -alrt 1000 -nt AUTO [78]. Branch support was assessed using 1000 ultrafast bootstrap replicates and SH-aLRT tests. Positive selection analysis was conducted using the aBSREL (adaptive Branch-Site Random Effects Likelihood) [89] (adaptive branch-site random effects likelihood) method implemented in HyPhy (v2.5.86, https://github.com/veg/hyphy). The analysis was performed with the following command and parameters: the aBSREL method tests whether individual branches in the phylogeny have experienced positive selection without requiring a prior specification of foreground branches. The model allows the distribution of ω (*dN/dS*) ratios to vary across both sites and branches, identifying episodic positive selection events. Significance was determined using likelihood ratio tests with FDR correction for multiple testing (*q*-value <0.05). All statistical analyses and visualizations were performed in R (v4.4.1) using the following packages: *ggtree*, *treeio*, *ggplot2*, and *dplyr*. aBSREL JSON output files (~50 MB) were parsed using optimized memory-efficient functions to handle large datasets.

### Synteny analysis of *PIP2*/*TIP2* genes in Rosaceae

The genomic data used for synteny analysis included partial species of Rosaceae (retrieved from the GDR database) and *Z. mauritiana* from a closely related family [83, 90]. Synteny analysis was performed using the mscan module in JCVI [87]. First, pairwise synteny searches were conducted with LASTAL based on the phylogenetic relationships of the species [91]. Subsequently, the *PIP2* and *TIP2* genes of OB and BT were selected to identify syntenic regions.

### *Cis*-regulatory element and expression analysis

Promoter regions (2000 bp upstream of ATG) from 38 *RcAQP*s and 37 *RwAQP*s were analyzed via PlantCARE [92]. Remove rows where the TATA box, CAAT box, and the names of *cis* elements were empty or unnamed. Then *cis* elements were summarized and visualized using *ggplot2* in *R* (v4.3.2). Transcriptome data from prickles and the cortex tissue below the prickles (CP) *R. gigantea* were explored from the Genome Sequence Archive of the National Genomics Data Center, China National Center for Bioinformation/Beijing Institute of Genomics, Chinese Academy of Sciences (GSA: CRA022471) [93]*.* Fragments Per Kilobase of exon model per Million mapped fragments (FPKM) normalization was utilized to assess the transcript abundance of *AQP* genes. Heatmaps were generated in TBtools following log_2_(FPKM+1) transformation with row standardization.

### Virus-induced gene silencing

A 291-bp segment of *RcPIP2* (*RchChr3g0469531*) was amplified by PCR and inserted into the *TRV2* vector to generate the *pTRV2-RcPIP2* construct [94]. Recombinant vectors (*pTRV1*, *pTRV2*, *pTRV2-RcPIP2*) were transformed into *Agrobacterium tumefaciens* GV3101. Bacterial cultures (OD600 = 1.0) containing *pTRV1* and *TRV-RcPIP2/pTRV2* at a ratio of 1:1 (v/v) in infiltration buffer (4.4 g/l MS salts, 30 g/l glucose, 200 μM acetosyringone) were vacuum-infiltrated (−0.06 kPa, 15 min) into plantlets. The plantlets were removed and wrapped in paper and placed in bags to keep moisture in the dark at 21°C overnight. After 1 week, DNA from new leaves was extracted and positive plantlets were screened by PCR. About 45 days later, RNA was extracted from leaves of newly formed second internode of plants for Reverse Transcription Quantitative Polymerase Chain Reaction (RT-qPCR) to analyze the *PIP2* expression. Number of prickles on the stem of positive plantlets was counted and photographed by hand.

### Expression analysis via RT-qPCR

First-strand cDNA synthesis utilized NovoScript® Plus SuperMix (Novoprotein). Reactions (10 μl) contained 5 μl 2 × SYBR mix, 0.2 μl ROXII, 0.2 μM primers, and 4.4 μl template. Amplification parameters: 95°C/1 min; 40 cycles of 95°C/20 s, 60°C/1 min; melt curve analysis (95°C/15 s, 60°C/1 min, 95°C/15 s). Relative expression was calculated via 2^−ΔΔCT^ method normalized to *RcUBC* `following protocols previously used' [95, 96]. Primer sequences are provided in Table S4.

## Supplementary Material

Web_Material_uhag143

## Data Availability

Raw RNA-seq data for three stages of prickles and prickle cortex can be found at the Genome Sequence Archive of National Genomics Data Center, China National Center for Bioinformation/Beijing Institute of Genomics, Chinese Academy of Sciences (GSA: CRA022471).
