# Supplementary material for: Pan-plantae aquaporin evolution: insights into PIP2 as a potential regulator of rose prickle development
Source: Hortic Res. 2026 Mar 14;13(8):uhag143. doi: 10.1093/hr/uhag143 (PMC13454484; doi:10.1093/hr/uhag143)

Figure S1

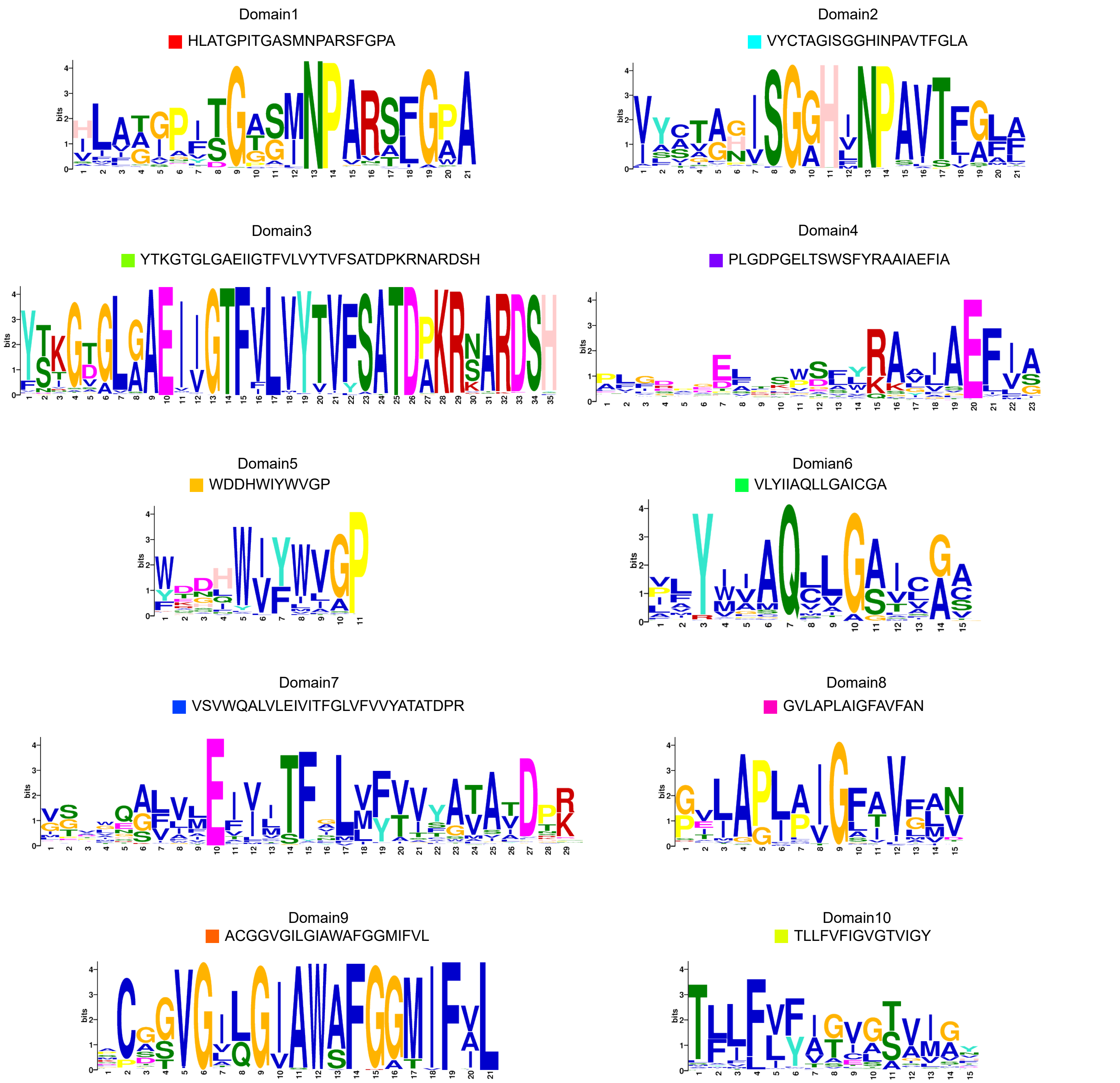
Domain1
HLATGPITGASMNPARSFGPA
Domain2
VYCTAGISGGHINPAVTFGLA
Domain3
YTKGTGLGAEIIGTFVLVYTVFSATDPKRNARDSH
Domain4
PLGDPGELTSWSFYRAAIAEFIA
Domain5
WDDHWIYWVGP
Domian6
VLYIIAQLLGAICGA
Domain7
VSVWQALVLEIVITFGLVFVVYATATDPR
Domain8
GVLAPLAIGFAVFAN
Domain9
ACGGVGILGIAWAFGGMIFVL
Domain10
TLLFVFIGVGTVIGY
bits

Figure S2

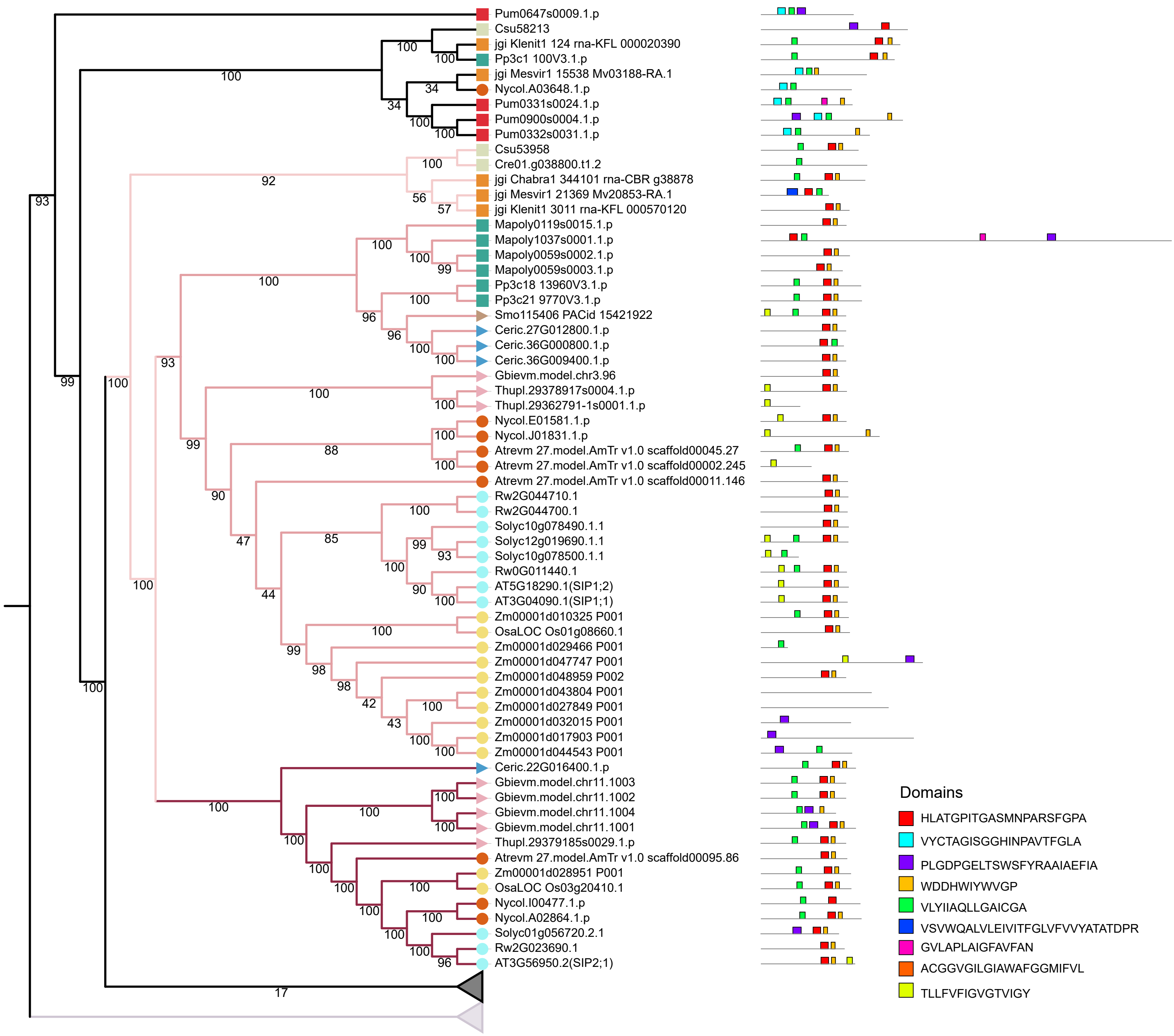

Pum0647s0009.1.p
Csu58213
jgi Klenit1 124 rna-KFL 000020390
Pp3c1 100V3.1.p
jgi Mesvir1 15538 Mv03188-RA.1
Nycol.A03648.1.p
Pum0331s0024.1.p
Pum0900s0004.1.p
Pum0332s0031.1.p
Csu53958
Cre01.g038800.t1.2
jgi Chabra1 344101 rna-CBR g38878
jgi Mesvir1 21369 Mv20853-RA.1
jgi Klenit1 3011 rna-KFL 000570120
Mapoly0119s0015.1.p
Mapoly1037s0001.1.p
Mapoly0059s0002.1.p
Mapoly0059s0003.1.p
Pp3c18 13960V3.1.p
Pp3c21 9770V3.1.p
Smo115406 PACid 15421922
Ceric.27G012800.1.p
Ceric.36G000800.1.p
Ceric.36G009400.1.p
Gbievm.model.chr3.96
Thupl.29378917s0004.1.p
Thupl.29362791-1s0001.1.p
Nycol.E01581.1.p
Nycol.J01831.1.p
Atrevm 27.model.AmTr v1.0 scaffold00045.27
Atrevm 27.model.AmTr v1.0 scaffold00002.245
Atrevm 27.model.AmTr v1.0 scaffold00011.146
Rw2G044710.1
Rw2G044700.1
Solyc10g078490.1.1
Solyc12g019690.1.1
Solyc10g078500.1.1
Rw0G011440.1
AT5G18290.1(SIP1;2)
AT3G04090.1(SIP1;1)
Zm00001d010325 P001
OsaLOC Os01g08660.1
Zm00001d029466 P001
Zm00001d047747 P001
Zm00001d048959 P002
Zm00001d043804 P001
Zm00001d027849 P001
Zm00001d032015 P001
Zm00001d017903 P001
Zm00001d044543 P001
Ceric.22G016400.1.p
Gbievm.model.chr11.1003
Gbievm.model.chr11.1002
Gbievm.model.chr11.1004
Gbievm.model.chr11.1001
Thupl.29379185s0029.1.p
Atrevm 27.model.AmTr v1.0 scaffold00095.86
Zm00001d028951 P001
OsaLOC Os03g20410.1
Nycol.I00477.1.p
Nycol.A02864.1.p
Solyc01g056720.2.1
Rw2G023690.1
AT3G56950.2(SIP2;1)
Domains
HLATGPITGASMNPARSFGPA
VYCTAGISGGHINPAVTFGLA
PLGDPGELTSWSFYRAAIAEFIA
WDDHWIYWVGP
VLYIIAQLLGAICGA
VSVWQALVLEIVITFGLVFVVYATATDPR
GVLAPLAIGFAVFAN
ACGGVGILGIAWAFGGMIFVL
TLLFVFIGVGTVIGY

Figure S3

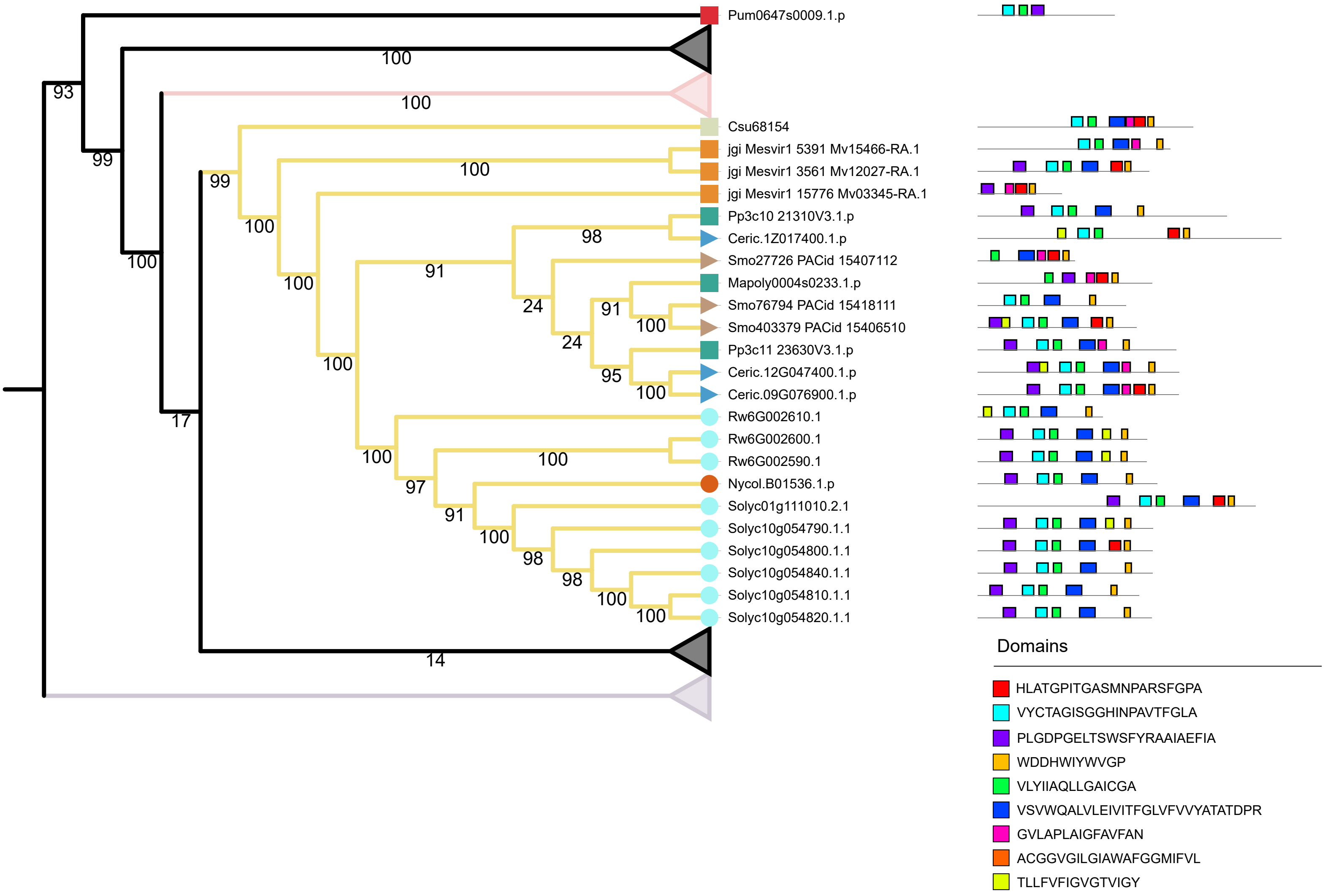

Pum0647s0009.1.p
Csu68154
jgi Mesvir1 5391 Mv15466-RA.1
jgi Mesvir1 3561 Mv12027-RA.1
jgi Mesvir1 15776 Mv03345-RA.1
Pp3c10 21310V3.1.p
Ceric.1Z017400.1.p
Smo27726 PACid 15407112
Mapoly0004s0233.1.p
Smo76794 PACid 15418111
Smo403379 PACid 15406510
Pp3c11 23630V3.1.p
Ceric.12G047400.1.p
Ceric.09G076900.1.p
Rw6G002610.1
Rw6G002600.1
Rw6G002590.1
Nycol.B01536.1.p
Solyc01g111010.2.1
Solyc10g054790.1.1
Solyc10g054800.1.1
Solyc10g054840.1.1
Solyc10g054810.1.1
Solyc10g054820.1.1
Domains
HLATGPITGASMNPARSFGPA
VYCTAGISGGHINPAVTFGLA
PLGDPGELTSWSFYRAAIAEFIA
WDDHWIYWVGP
VLYIIAQLLGAICGA
VSVWQALVLEIVITFGLVFVVYATATDPR
GVLAPLAIGFAVFAN
ACGGVGILGIAWAFGGMIFVL
TLLFVFIGVGTVIGY

Figure S4

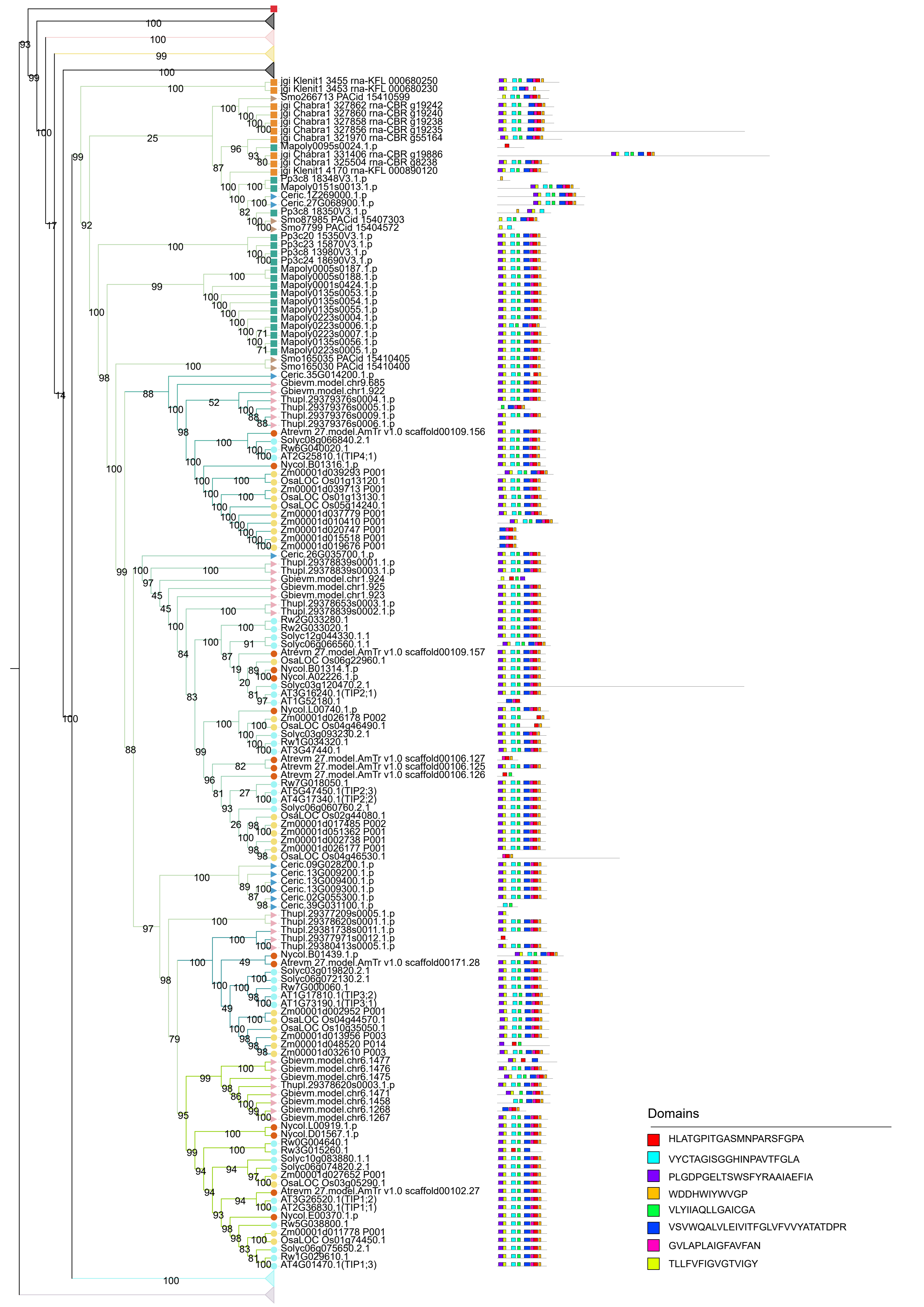
jgi Klenit1 3455 rna-KFL 000680250
jgi Klenit1 3453 rna-KFL 000680230
Smo266713 PACid 15410599
jgi Chabra1 327862 rna-CBR g19242
jgi Chabra1 327860 rna-CBR g19240
jgi Chabra1 327858 rna-CBR g19238
jgi Chabra1 327856 rna-CBR g19235
jgi Chabra1 321970 rna-CBR g55164
Mapoly0095s0024.1.p
jgi Chabra1 331406 rna-CBR g19886
jgi Chabra1 325504 rna-CBR g8238
jgi Klenit1 4170 rna-KFL 000890120
Pp3c8 18348V3.1.p
Mapoly0151s0013.1.p
Ceric.1Z269000.1.p
Ceric.27G068900.1.p
Pp3c8 18350V3.1.p
Smo87985 PACid 15407303
Smo7799 PACid 15404572
Pp3c20 15350V3.1.p
Pp3c23 15870V3.1.p
Pp3c8 13980V3.1.p
Pp3c24 18690V3.1.p
Mapoly0005s0187.1.p
Mapoly0005s0188.1.p
Mapoly0001s0424.1.p
Mapoly0135s0053.1.p
Mapoly0135s0054.1.p
Mapoly0135s0055.1.p
Mapoly0223s0004.1.p
Mapoly0223s0006.1.p
Mapoly0223s0007.1.p
Mapoly0135s0056.1.p
Mapoly0223s0005.1.p
Smo165035 PACid 15410405
Smo165030 PACid 15410400
Ceric.35G014200.1.p
Gbievm.model.chr9.685
Gbievm.model.chr1.922
Thupl.29379376s0004.1.p
Thupl.29379376s0005.1.p
Thupl.29379376s0009.1.p
Thupl.29379376s0006.1.p
Atrevm 27.model.AmTr v1.0 scaffold00109.156
Solyc08g066840.2.1
Rw6G040020.1
AT2G25810.1(TIP4;1)
Nycol.B01316.1.p
Zm00001d039293 P001
OsaLOC Os01g13120.1
Zm00001d039713 P001
OsaLOC Os01g13130.1
OsaLOC Os05g14240.1
Zm00001d037779 P001
Zm00001d010410 P001
Zm00001d020747 P001
Zm00001d015518 P001
Zm00001d019676 P001
Ceric.26G035700.1.p
Thupl.29378839s0001.1.p
Thupl.29378839s0003.1.p
Gbievm.model.chr1.924
Gbievm.model.chr1.925
Gbievm.model.chr1.923
Thupl.29378653s0003.1.p
Thupl.29378839s0002.1.p
Rw2G033280.1
Rw2G033020.1
Solyc12g044330.1.1
Solyc06g066560.1.1
Atrevm 27.model.AmTr v1.0 scaffold00109.157
OsaLOC Os06g22960.1
Nycol.B01314.1.p
Nycol.A02226.1.p
Solyc03g120470.2.1
AT3G16240.1(TIP2;1)
AT1G52180.1
Nycol.L00740.1.p
Zm00001d026178 P002
OsaLOC Os04g46490.1
Solyc03g093230.2.1
Rw1G034320.1
AT3G47440.1
Atrevm 27.model.AmTr v1.0 scaffold00106.127
Atrevm 27.model.AmTr v1.0 scaffold00106.125
Atrevm 27.model.AmTr v1.0 scaffold00106.126
Rw7G018050.1
AT5G47450.1(TIP2;3)
AT4G17340.1(TIP2;2)
Solyc06g060760.2.1
OsaLOC Os02g44080.1
Zm00001d017485 P002
Zm00001d051362 P001
Zm00001d002738 P001
Zm00001d026177 P001
OsaLOC Os04g46530.1
Ceric.09G028200.1.p
Ceric.13G009200.1.p
Ceric.13G009400.1.p
Ceric.13G009300.1.p
Ceric.02G055300.1.p
Ceric.39G031100.1.p
Thupl.29377209s0005.1.p
Thupl.29378620s0001.1.p
Thupl.29381738s0011.1.p
Thupl.29377971s0012.1.p
Thupl.29380413s0005.1.p
Nycol.B01439.1.p
Atrevm 27.model.AmTr v1.0 scaffold00171.28
Solyc03g019820.2.1
Solyc06g072130.2.1
Rw7G000060.1
AT1G17810.1(TIP3;2)
AT1G73190.1(TIP3;1)
Zm00001d002952 P001
OsaLOC Os04g44570.1
OsaLOC Os10g35050.1
Zm00001d013956 P003
Zm00001d048520 P014
Zm00001d032610 P003
Gbievm.model.chr6.1477
Gbievm.model.chr6.1476
Gbievm.model.chr6.1475
Thupl.29378620s0003.1.p
Gbievm.model.chr6.1471
Gbievm.model.chr6.1458
Gbievm.model.chr6.1268
Gbievm.model.chr6.1267
Nycol.L00919.1.p
Nycol.D01567.1.p
Rw0G004640.1
Rw3G015260.1
Solyc10g083880.1.1
Solyc06g074820.2.1
Zm00001d027652 P001
OsaLOC Os03g05290.1
Atrevm 27.model.AmTr v1.0 scaffold00102.27
AT3G26520.1(TIP1;2)
AT2G36830.1(TIP1;1)
Nycol.E00370.1.p
Rw5G038800.1
Zm00001d011778 P001
OsaLOC Os01g74450.1
Solyc06g075650.2.1
Rw1G029610.1
AT4G01470.1(TIP1;3)
Domains
HLATGPITGASMNPARSFGPA
VYCTAGISGGHINPAVTFGLA
PLGDPGELTSWSFYRAAIAEFIA
WDDHWIYWVGP
VLYIIAQLLGAICGA
VSVWQALVLEIVITFGLVFVVYATATDPR
GVLAPLAIGFAVFAN
TLLFVFIGVGTVIGY

Figure S5

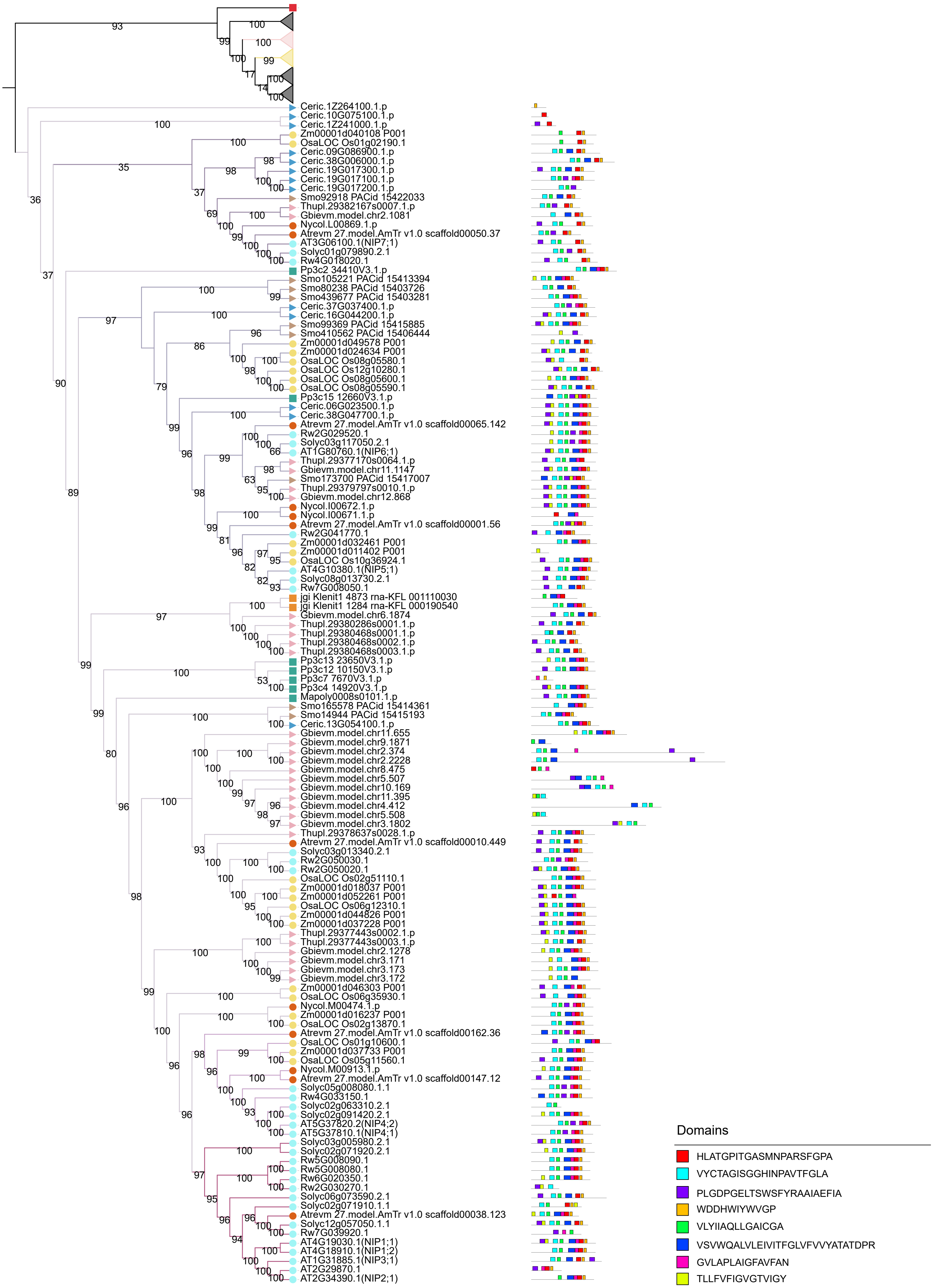
Ceric.1Z264100.1.p
Ceric.10G075100.1.p
Ceric.1Z241000.1.p
Zm00001d040108 P001
OsaLOC Os01g02190.1
Ceric.09G086900.1.p
Ceric.38G006000.1.p
Ceric.19G017300.1.p
Ceric.19G017100.1.p
Ceric.19G017200.1.p
Smo92918 PACid 15422033
Thupl.29382167s0007.1.p
Gbievm.model.chr2.1081
Nycol.L00869.1.p
Atrevm 27.model.AmTr v1.0 scaffold00050.37
AT3G06100.1(NIP7;1)
Solyc01g079890.2.1
Rw4G018020.1
Pp3c2 34410V3.1.p
Smo105221 PACid 15413394
Smo80238 PACid 15403726
Smo439677 PACid 15403281
Ceric.37G037400.1.p
Ceric.16G044200.1.p
Smo99369 PACid 15415885
Smo410562 PACid 15406444
Zm00001d049578 P001
Zm00001d024634 P001
OsaLOC Os08g05580.1
OsaLOC Os12g10280.1
OsaLOC Os08g05600.1
OsaLOC Os08g05590.1
Pp3c15 12660V3.1.p
Ceric.06G023500.1.p
Ceric.38G047700.1.p
Atrevm 27.model.AmTr v1.0 scaffold00065.142
Rw2G029520.1
Solyc03g117050.2.1
AT1G80760.1(NIP6;1)
Thupl.29377170s0064.1.p
Gbievm.model.chr11.1147
Smo173700 PACid 15417007
Thupl.29379797s0010.1.p
Gbievm.model.chr12.868
Nycol.I00672.1.p
Nycol.I00671.1.p
Atrevm 27.model.AmTr v1.0 scaffold00001.56
Rw2G041770.1
Zm00001d032461 P001
Zm00001d011402 P001
OsaLOC Os10g36924.1
AT4G10380.1(NIP5;1)
Solyc08g013730.2.1
Rw7G008050.1
jgi Klenit1 4873 rna-KFL 001110030
jgi Klenit1 1284 rna-KFL 000190540
Gbievm.model.chr6.1874
Thupl.29380286s0001.1.p
Thupl.29380468s0001.1.p
Thupl.29380468s0002.1.p
Thupl.29380468s0003.1.p
Pp3c13 23650V3.1.p
Pp3c12 10150V3.1.p
Pp3c7 7670V3.1.p
Pp3c4 14920V3.1.p
Mapoly0008s0101.1.p
Smo165578 PACid 15414361
Smo14944 PACid 15415193
Ceric.13G054100.1.p
Gbievm.model.chr11.655
Gbievm.model.chr9.1871
Gbievm.model.chr2.374
Gbievm.model.chr2.2228
Gbievm.model.chr8.475
Gbievm.model.chr5.507
Gbievm.model.chr10.169
Gbievm.model.chr11.395
Gbievm.model.chr4.412
Gbievm.model.chr5.508
Gbievm.model.chr3.1802
Thupl.29378637s0028.1.p
Atrevm 27.model.AmTr v1.0 scaffold00010.449
Solyc03g013340.2.1
Rw2G050030.1
Rw2G050020.1
OsaLOC Os02g51110.1
Zm00001d018037 P001
Zm00001d052261 P001
OsaLOC Os06g12310.1
Zm00001d044826 P001
Zm00001d037228 P001
Thupl.29377443s0002.1.p
Thupl.29377443s0003.1.p
Gbievm.model.chr2.1278
Gbievm.model.chr3.171
Gbievm.model.chr3.173
Gbievm.model.chr3.172
Zm00001d046303 P001
OsaLOC Os06g35930.1
Nycol.M00474.1.p
Zm00001d016237 P001
OsaLOC Os02g13870.1
Atrevm 27.model.AmTr v1.0 scaffold00162.36
OsaLOC Os01g10600.1
Zm00001d037733 P001
OsaLOC Os05g11560.1
Nycol.M00913.1.p
Atrevm 27.model.AmTr v1.0 scaffold00147.12
Solyc05g008080.1.1
Rw4G033150.1
Solyc02g063310.2.1
Solyc02g091420.2.1
AT5G37820.2(NIP4;2)
AT5G37810.1(NIP4;1)
Solyc03g005980.2.1
Solyc02g071920.2.1
Rw5G008090.1
Rw5G008080.1
Rw6G020350.1
Rw2G030270.1
Solyc06g073590.2.1
Solyc02g071910.1.1
Atrevm 27.model.AmTr v1.0 scaffold00038.123
Solyc12g057050.1.1
Rw7G039920.1
AT4G19030.1(NIP1;1)
AT4G18910.1(NIP1;2)
AT1G31885.1(NIP3;1)
AT2G29870.1
AT2G34390.1(NIP2;1)
Domains
HLATGPITGASMNPARSFGPA
VYCTAGISGGHINPAVTFGLA
PLGDPGELTSWSFYRAAIAEFIA
WDDHWIYWVGP
VLYIIAQLLGAICGA
VSVWQALVLEIVITFGLVFVVYATATDPR
GVLAPLAIGFAVFAN
TLLFVFIGVGTVIGY

Figure S6

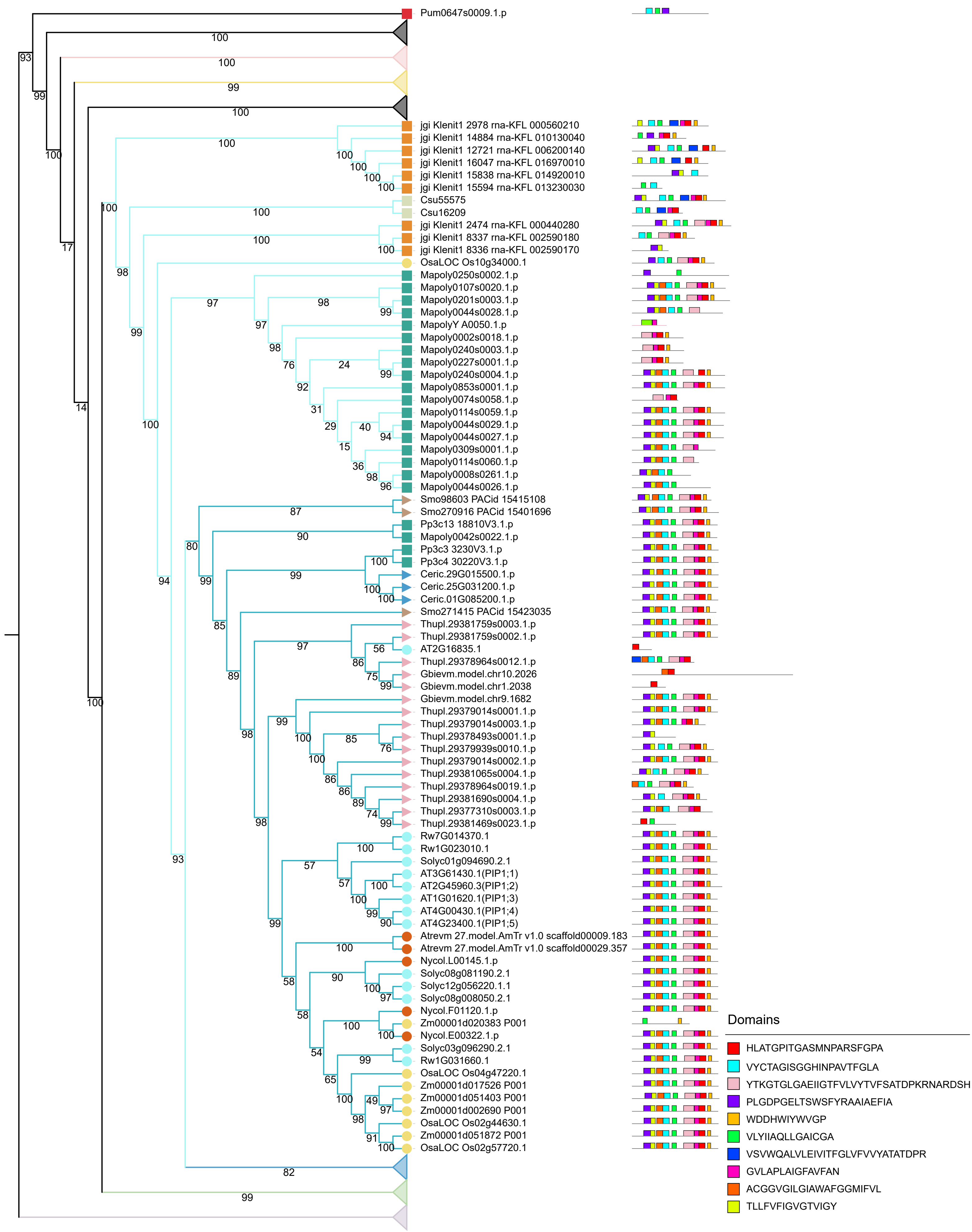

Pum0647s0009.1.p
jgi Klenit1 2978 rna-KFL 000560210
jgi Klenit1 14884 rna-KFL 010130040
jgi Klenit1 12721 rna-KFL 006200140
jgi Klenit1 16047 rna-KFL 016970010
jgi Klenit1 15838 rna-KFL 014920010
jgi Klenit1 15594 rna-KFL 013230030
Csu55575
Csu16209
jgi Klenit1 2474 rna-KFL 000440280
jgi Klenit1 8337 rna-KFL 002590180
jgi Klenit1 8336 rna-KFL 002590170
OsaLOC Os10g34000.1
Mapoly0250s0002.1.p
Mapoly0107s0020.1.p
Mapoly0201s0003.1.p
Mapoly0044s0028.1.p
MapolyY A0050.1.p
Mapoly0002s0018.1.p
Mapoly0240s0003.1.p
Mapoly0227s0001.1.p
Mapoly0240s0004.1.p
Mapoly0853s0001.1.p
Mapoly0074s0058.1.p
Mapoly0114s0059.1.p
Mapoly0044s0029.1.p
Mapoly0044s0027.1.p
Mapoly0309s0001.1.p
Mapoly0114s0060.1.p
Mapoly0008s0261.1.p
Mapoly0044s0026.1.p
Smo98603 PACid 15415108
Smo270916 PACid 15401696
Pp3c13 18810V3.1.p
Mapoly0042s0022.1.p
Pp3c3 3230V3.1.p
Pp3c4 30220V3.1.p
Ceric.29G015500.1.p
Ceric.25G031200.1.p
Ceric.01G085200.1.p
Smo271415 PACid 15423035
Thupl.29381759s0003.1.p
Thupl.29381759s0002.1.p
AT2G16835.1
Thupl.29378964s0012.1.p
Gbievm.model.chr10.2026
Gbievm.model.chr1.2038
Gbievm.model.chr9.1682
Thupl.29379014s0001.1.p
Thupl.29379014s0003.1.p
Thupl.29378493s0001.1.p
Thupl.29379939s0010.1.p
Thupl.29379014s0002.1.p
Thupl.29381065s0004.1.p
Thupl.29378964s0019.1.p
Thupl.29381690s0004.1.p
Thupl.29377310s0003.1.p
Thupl.29381469s0023.1.p
Rw7G014370.1
Rw1G023010.1
Solyc01g094690.2.1
AT3G61430.1(PIP1;1)
AT2G45960.3(PIP1;2)
AT1G01620.1(PIP1;3)
AT4G00430.1(PIP1;4)
AT4G23400.1(PIP1;5)
Atrevm 27.model.AmTr v1.0 scaffold00009.183
Atrevm 27.model.AmTr v1.0 scaffold00029.357
Nycol.L00145.1.p
Solyc08g081190.2.1
Solyc12g056220.1.1
Solyc08g008050.2.1
Nycol.F01120.1.p
Zm00001d020383 P001
Nycol.E00322.1.p
Solyc03g096290.2.1
Rw1G031660.1
OsaLOC Os04g47220.1
Zm00001d017526 P001
Zm00001d051403 P001
Zm00001d002690 P001
OsaLOC Os02g44630.1
Zm00001d051872 P001
OsaLOC Os02g57720.1
Domains
HLATGPITGASMNPARSFGPA
VYCTAGISGGHINPAVTFGLA
YTKGTGLGAEIIGTFVLVYTVFSATDPKRNARDSH
PLGDPGELTSWSFYRAAIAEFIA
WDDHWIYWVGP
VLYIIAQLLGAICGA
VSVWQALVLEIVITFGLVFVVYATATDPR
GVLAPLAIGFAVFAN
ACGGVGILGIAWAFGGMIFVL
TLLFVFIGVGTVIGY

Figure S7

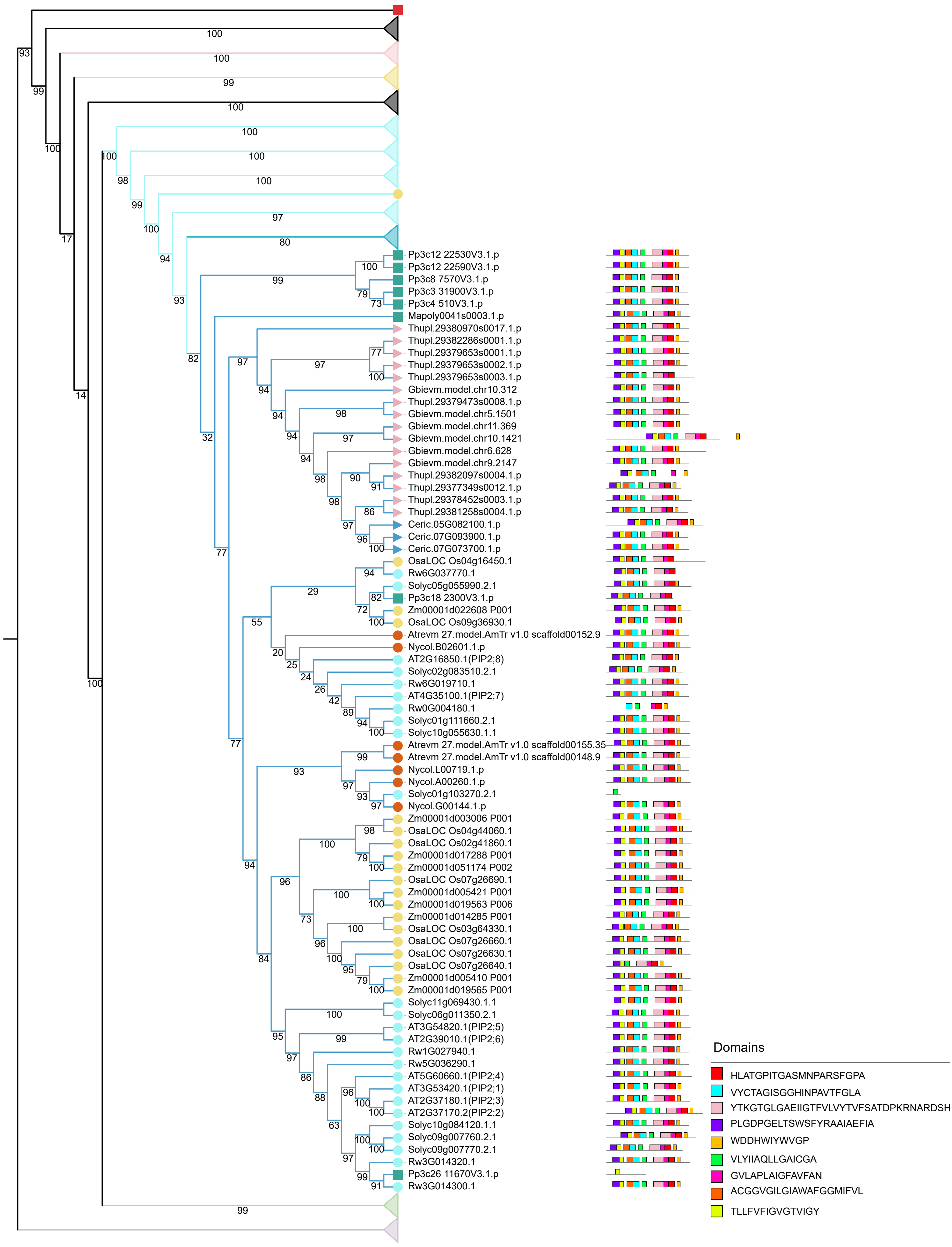
Pp3c12 22530V3.1.p
Pp3c12 22590V3.1.p
Pp3c8 7570V3.1.p
Pp3c3 31900V3.1.p
Pp3c4 510V3.1.p
Mapoly0041s0003.1.p
Thupl.29380970s0017.1.p
Thupl.29382286s0001.1.p
Thupl.29379653s0001.1.p
Thupl.29379653s0002.1.p
Thupl.29379653s0003.1.p
Gbievm.model.chr10.312
Thupl.29379473s0008.1.p
Gbievm.model.chr5.1501
Gbievm.model.chr11.369
Gbievm.model.chr10.1421
Gbievm.model.chr6.628
Gbievm.model.chr9.2147
Thupl.29382097s0004.1.p
Thupl.29377349s0012.1.p
Thupl.29378452s0003.1.p
Thupl.29381258s0004.1.p
Ceric.05G082100.1.p
Ceric.07G093900.1.p
Ceric.07G073700.1.p
OsaLOC Os04g16450.1
Rw6G037770.1
Solyc05g055990.2.1
Pp3c18 2300V3.1.p
Zm00001d022608 P001
OsaLOC Os09g36930.1
Atrevm 27.model.AmTr v1.0 scaffold00152.9
Nycol.B02601.1.p
AT2G16850.1(PIP2;8)
Solyc02g083510.2.1
Rw6G019710.1
AT4G35100.1(PIP2;7)
Rw0G004180.1
Solyc01g111660.2.1
Solyc10g055630.1.1
Atrevm 27.model.AmTr v1.0 scaffold00155.35
Atrevm 27.model.AmTr v1.0 scaffold00148.9
Nycol.L00719.1.p
Nycol.A00260.1.p
Solyc01g103270.2.1
Nycol.G00144.1.p
Zm00001d003006 P001
OsaLOC Os04g44060.1
OsaLOC Os02g41860.1
Zm00001d017288 P001
Zm00001d051174 P002
OsaLOC Os07g26690.1
Zm00001d005421 P001
Zm00001d019563 P006
Zm00001d014285 P001
OsaLOC Os03g64330.1
OsaLOC Os07g26660.1
OsaLOC Os07g26630.1
OsaLOC Os07g26640.1
Zm00001d005410 P001
Zm00001d019565 P001
Solyc11g069430.1.1
Solyc06g011350.2.1
AT3G54820.1(PIP2;5)
AT2G39010.1(PIP2;6)
Rw1G027940.1
Rw5G036290.1
AT5G60660.1(PIP2;4)
AT3G53420.1(PIP2;1)
AT2G37180.1(PIP2;3)
AT2G37170.2(PIP2;2)
Solyc10g084120.1.1
Solyc09g007760.2.1
Solyc09g007770.2.1
Rw3G014320.1
Pp3c26 11670V3.1.p
Rw3G014300.1
Domains
HLATGPITGASMNPARSFGPA
VYCTAGISGGHINPAVTFGLA
YTKGTGLGAEIIGTFVLVYTVFSATDPKRNARDSH
PLGDPGELTSWSFYRAAIAEFIA
WDDHWIYWVGP
VLYIIAQLLGAICGA
GVLAPLAIGFAVFAN
ACGGVGILGIAWAFGGMIFVL
TLLFVFIGVGTVIGY

Figure S8

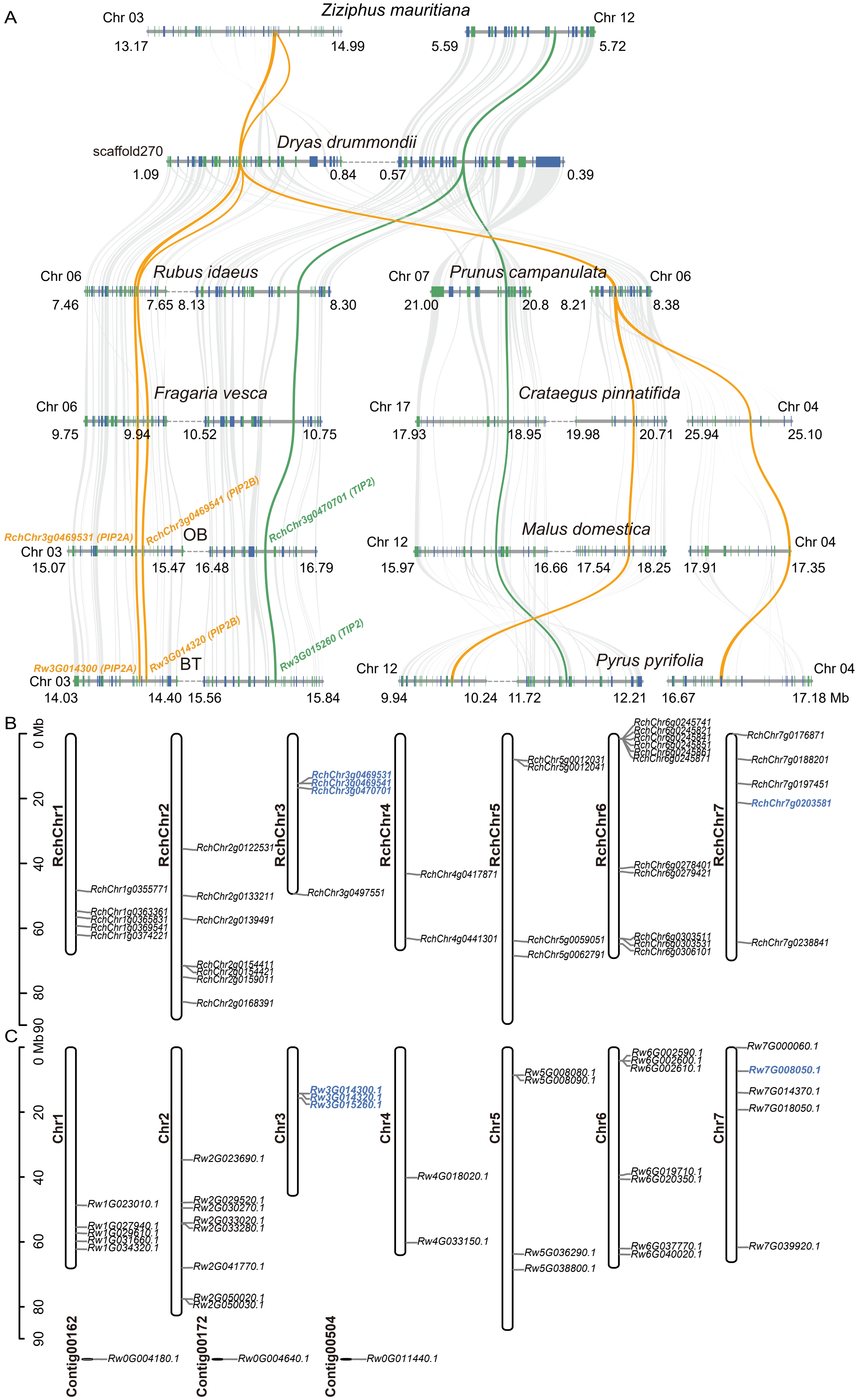
A
Ziziphus mauritiana
Chr 03
13.17
14.99
5.59
Chr 12
5.72
Dryas drummondii
scaffold270
1.09
0.84
0.57
0.39
Rubus idaeus
Chr 06
7.46
7.65
8.13
8.30
Chr 07
Prunus campanulata
Chr 06
21.00
20.8
8.21
8.38
Fragaria vesca
Chr 06
9.75
9.94
10.52
10.75
Chr 17
Crataegus pinnatifida
Chr 04
17.93
18.95
19.98
20.71
25.94
25.10
RchChr3g0469531 (PIP2A)
RchChr3g0469541 (PIP2B)
RchChr3g0470701 (TIP2)
OB
Chr 03
15.07
15.47
16.48
16.79
Malus domestica
Chr 12
Chr 04
15.97
16.66
17.54
18.25
17.91
17.35
Rw3G014300 (PIP2A)
Rw3G014320 (PIP2B)
Rw3G015260 (TIP2)
BT
Chr 03
14.03
14.40
15.56
15.84
Pyrus pyrifolia
Chr 12
Chr 04
9.94
10.24
11.72
12.21
16.67
17.18 Mb
B
0 Mb
20
40
60
80
90
RchChr1
RchChr1g0355771
RchChr1g0363361
RchChr1g0365831
RchChr1g0369541
RchChr1g0374221
RchChr2
RchChr2g0122531
RchChr2g0133211
RchChr2g0139491
RchChr2g0154411
RchChr2g0154421
RchChr2g0159011
RchChr2g0168391
RchChr3
RchChr3g0469531
RchChr3g0469541
RchChr3g0470701
RchChr3g0497551
RchChr4
RchChr4g0417871
RchChr4g0441301
RchChr5
RchChr5g0012031
RchChr5g0012041
RchChr5g0059051
RchChr5g0062791
RchChr6
RchChr6g0245741
RchChr6g0245821
RchChr6g0245841
RchChr6g0245851
RchChr6g0245861
RchChr6g0245871
RchChr6g0278401
RchChr6g0279421
RchChr6g0303511
RchChr6g0303531
RchChr6g0306101
RchChr7
RchChr7g0176871
RchChr7g0188201
RchChr7g0197451
RchChr7g0203581
RchChr7g0238841
C
0 Mb
20
40
60
80
90
Chr1
Rw1G023010.1
Rw1G027940.1
Rw1G029610.1
Rw1G031660.1
Rw1G034320.1
Chr2
Rw2G023690.1
Rw2G029520.1
Rw2G030270.1
Rw2G033020.1
Rw2G033280.1
Rw2G041770.1
Rw2G050020.1
Rw2G050030.1
Chr3
Rw3G014300.1
Rw3G014320.1
Rw3G015260.1
Chr4
Rw4G018020.1
Rw4G033150.1
Chr5
Rw5G008080.1
Rw5G008090.1
Rw5G036290.1
Rw5G038800.1
Chr6
Rw6G002590.1
Rw6G002600.1
Rw6G002610.1
Rw6G019710.1
Rw6G020350.1
Rw6G037770.1
Rw6G040020.1
Chr7
Rw7G000060.1
Rw7G008050.1
Rw7G014370.1
Rw7G018050.1
Rw7G039920.1
Contig00162
Rw0G004180.1
Contig00172
Rw0G004640.1
Contig00504
Rw0G011440.1

Figure S9

A

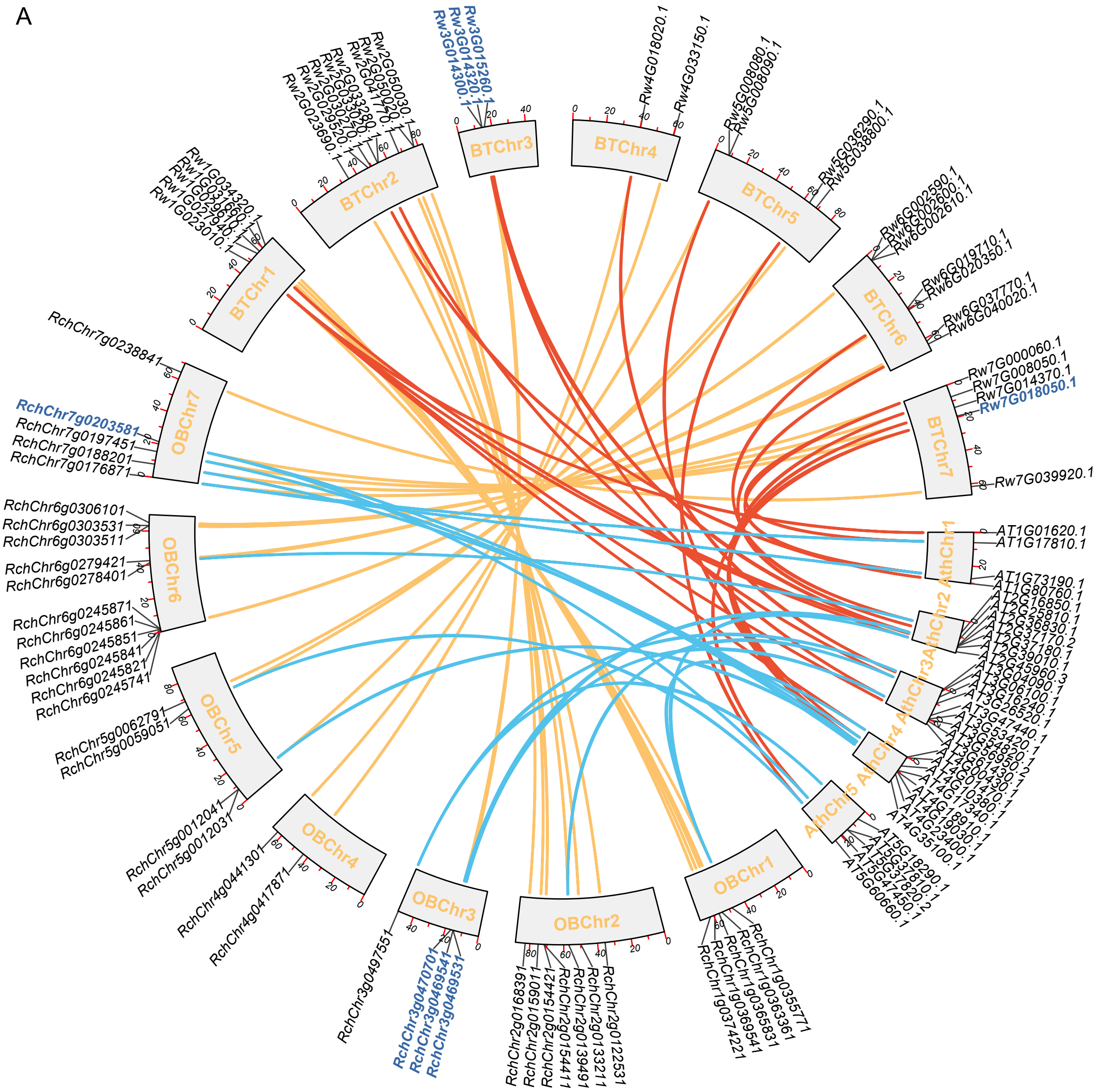

BTChr1
BTChr2
BTChr3
BTChr4
BTChr5
BTChr6
BTChr7
AthChr1
AthChr2
AthChr3
AthChr4
AthChr5
OBChr1
OBChr2
OBChr3
OBChr4
OBChr5
OBChr6
OBChr7

B

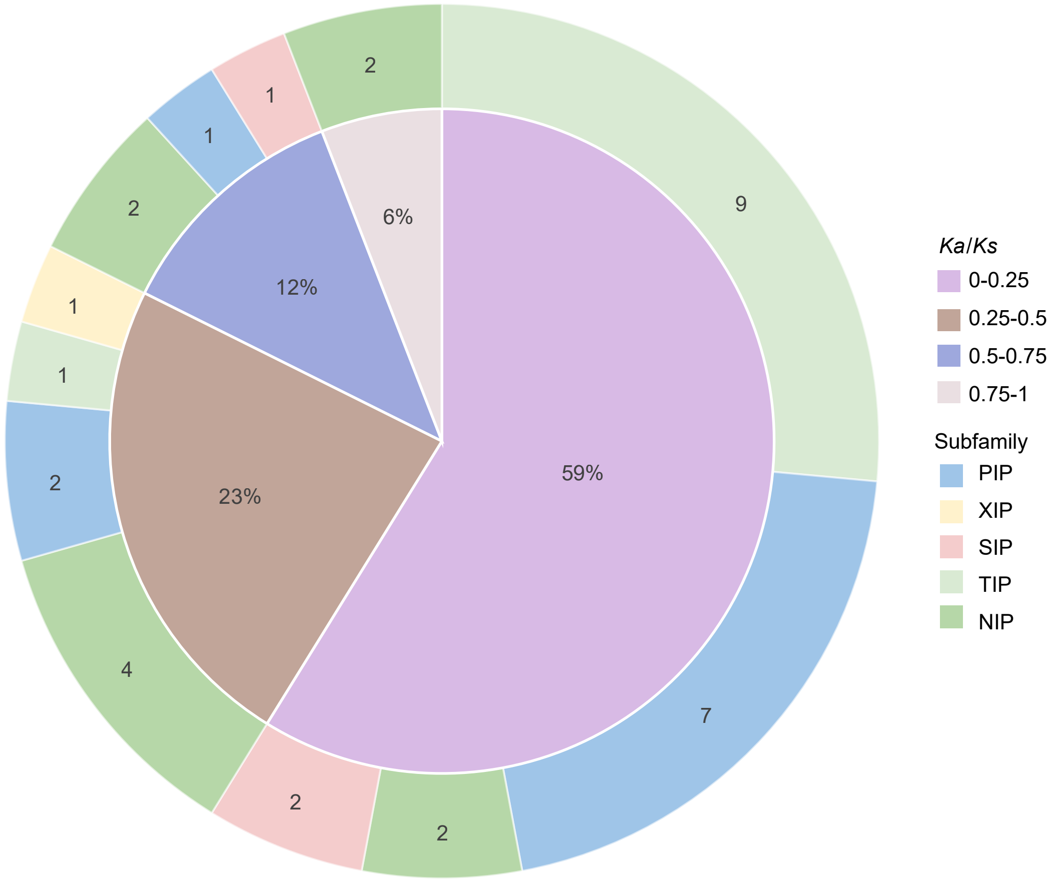

59%
23%
12%
6%
9
7
2
2
4
2
1
1
2
1
1
2
Ka/Ks
0-0.25
0.25-0.5
0.5-0.75
0.75-1
Subfamily
PIP
XIP
SIP
TIP
NIP

Figure S10

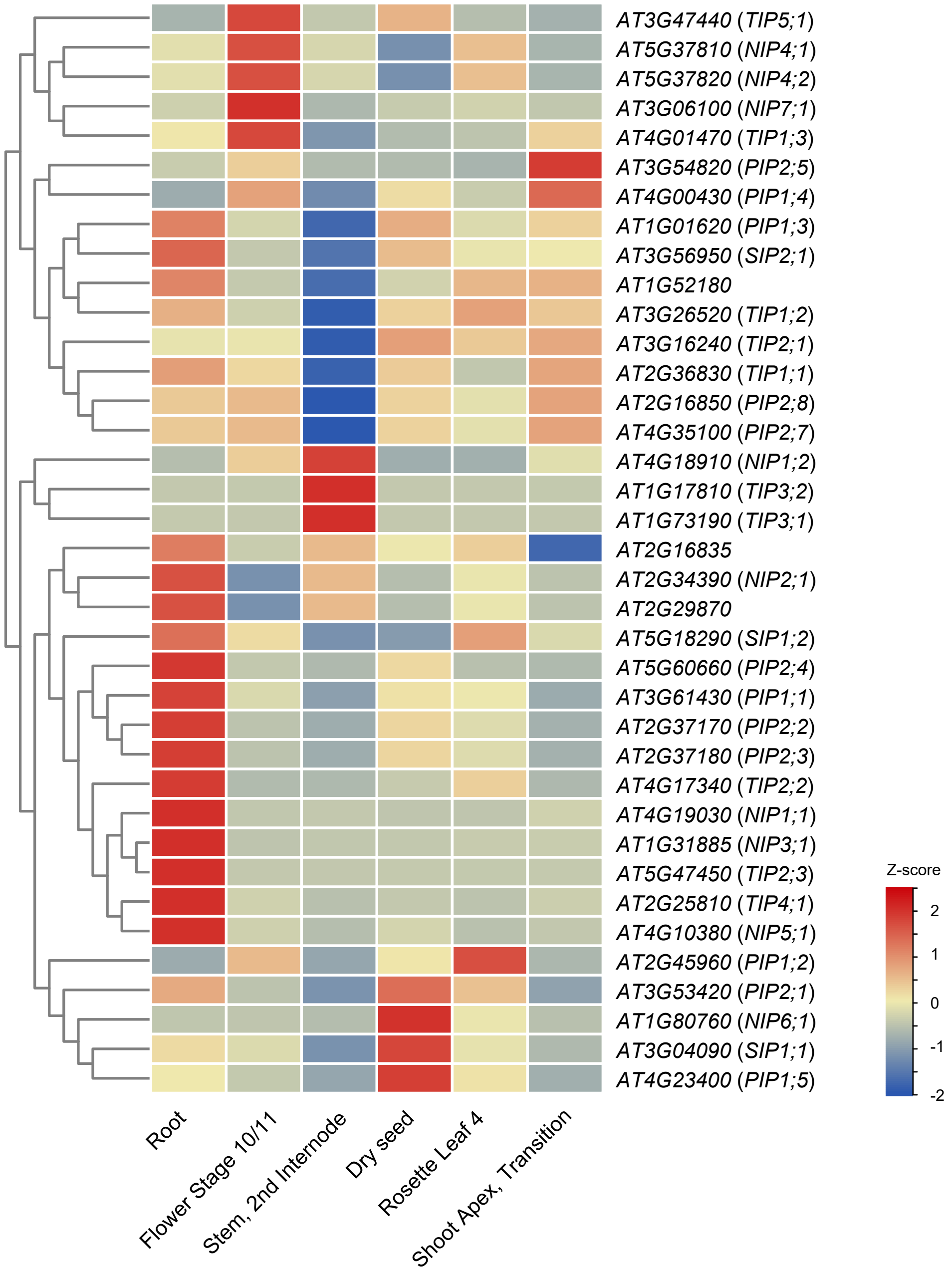
AT3G47440 (TIP5;1)
AT5G37810 (NIP4;1)
AT5G37820 (NIP4;2)
AT3G06100 (NIP7;1)
AT4G01470 (TIP1;3)
AT3G54820 (PIP2;5)
AT4G00430 (PIP1;4)
AT1G01620 (PIP1;3)
AT3G56950 (SIP2;1)
AT1G52180
AT3G26520 (TIP1;2)
AT3G16240 (TIP2;1)
AT2G36830 (TIP1;1)
AT2G16850 (PIP2;8)
AT4G35100 (PIP2;7)
AT4G18910 (NIP1;2)
AT1G17810 (TIP3;2)
AT1G73190 (TIP3;1)
AT2G16835
AT2G34390 (NIP2;1)
AT2G29870
AT5G18290 (SIP1;2)
AT5G60660 (PIP2;4)
AT3G61430 (PIP1;1)
AT2G37170 (PIP2;2)
AT2G37180 (PIP2;3)
AT4G17340 (TIP2;2)
AT4G19030 (NIP1;1)
AT1G31885 (NIP3;1)
AT5G47450 (TIP2;3)
AT2G25810 (TIP4;1)
AT4G10380 (NIP5;1)
AT2G45960 (PIP1;2)
AT3G53420 (PIP2;1)
AT1G80760 (NIP6;1)
AT3G04090 (SIP1;1)
AT4G23400 (PIP1;5)
Root
Flower Stage 10/11
Stem, 2nd Internode
Dry seed
Rosette Leaf 4
Shoot Apex, Transition
Z-score
2
1
0
-1
-2

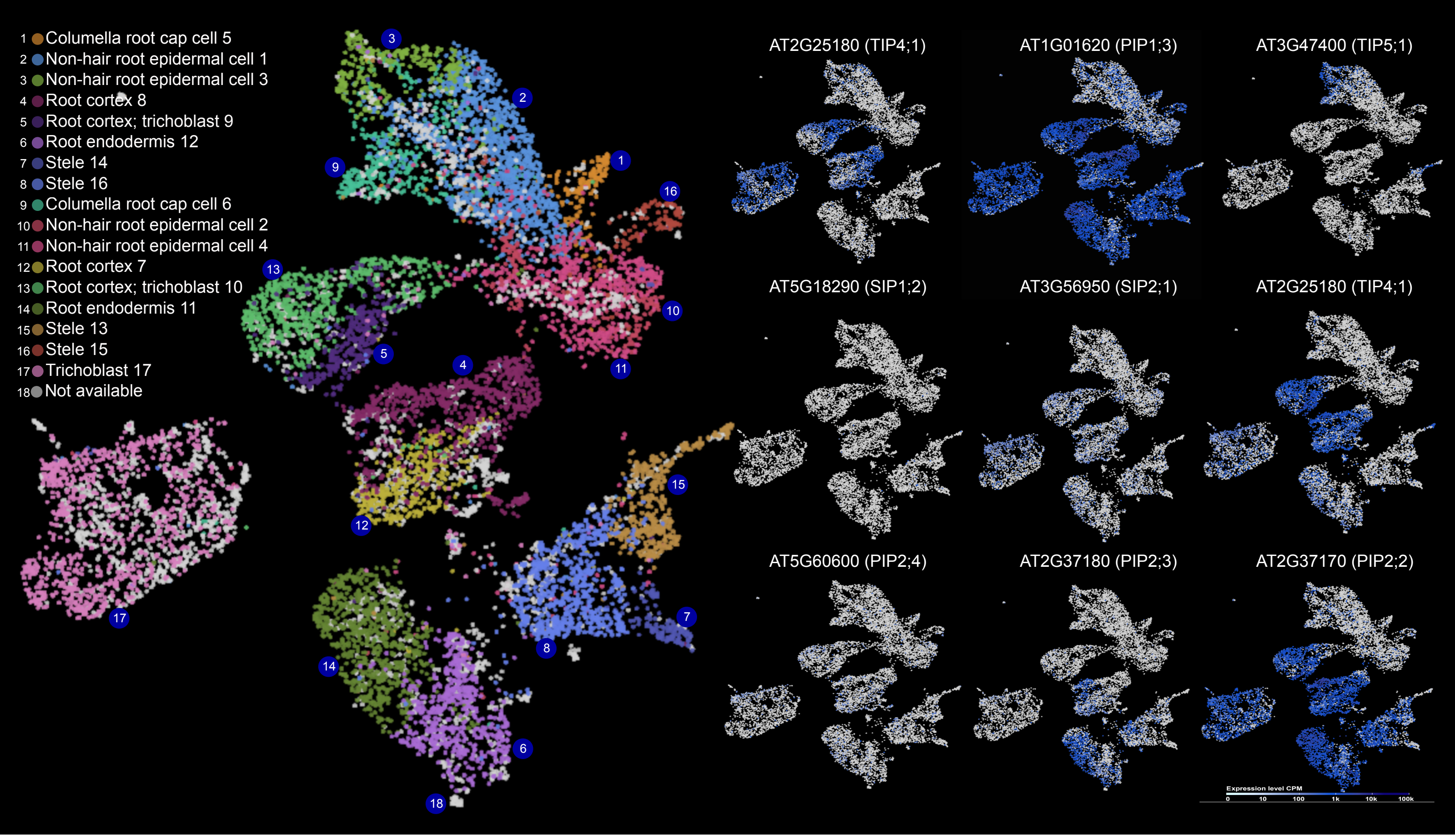
1 Columella root cap cell 5
2 Non-hair root epidermal cell 1
3 Non-hair root epidermal cell 3
4 Root cortex 8
5 Root cortex; trichoblast 9
6 Root endodermis 12
7 Stele 14
8 Stele 16
9 Columella root cap cell 6
10 Non-hair root epidermal cell 2
11 Non-hair root epidermal cell 4
12 Root cortex 7
13 Root cortex; trichoblast 10
14 Root endodermis 11
15 Stele 13
16 Stele 15
17 Trichoblast 17
18 Not available
AT2G25180 (TIP4;1)
AT1G01620 (PIP1;3)
AT3G47400 (TIP5;1)
AT5G18290 (SIP1;2)
AT3G56950 (SIP2;1)
AT2G25180 (TIP4;1)
AT5G60600 (PIP2;4)
AT2G37180 (PIP2;3)
AT2G37170 (PIP2;2)

Figure S12

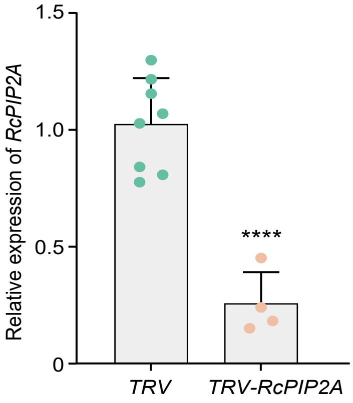

Supplement: Supplementary file 1 [file web_material_uhag143.zip › FigS1_S12_merged.pdf]
