# Supplementary material for: Pan-plantae aquaporin evolution: insights into PIP2 as a potential regulator of rose prickle development
Source: Hortic Res. 2026 Mar 14;13(8):uhag143. doi: 10.1093/hr/uhag143 (PMC13454484; doi:10.1093/hr/uhag143)
Supplement: Supplementary file 1 [file web_material_uhag143.zip › Table S1-S4.pdf]

Table S1. Protein structure, phosphorylation sites and subcellular localization information for 37 RwAQPs.

	Sequence ID	Number of AA	MW	PI	No. of Phosphor Sites	Prediction for cellular-localization
PIP	Rw0G004180.1	240	25946.19	7	Ser: 6 Thr: 3 Tyr: 4	chloroplast
	Rw1G023010.1	287	30869.73	8.47	Ser: 4 Thr: 8 Tyr: 4	plasma
	Rw1G027940.1	283	30153.99	8.44	Ser: 16 Thr: 9 Tyr: 3	plasma
	Rw1G031660.1	290	31097.31	8.89	Ser: 8 Thr: 10 Tyr: 3	plasma
	Rw3G014300.1	284	30006.66	8.29	Ser: 11 Thr: 8 Tyr: 3	plasma
	Rw3G014320.1	284	30031.84	8.28	Ser: 10 Thr: 8 Tyr: 3	plasma
	Rw5G036290.1	281	30195.11	7.05	Ser: 10 Thr: 7 Tyr: 4	plasma
	Rw6G019710.1	280	29964.85	8.99	Ser: 10 Thr: 5 Tyr: 3	plasma
	Rw6G037770.1	271	28578.06	6.37	Ser: 12 Thr: 7 Tyr: 4	plasma
	Rw7G014370.1	286	30887.97	8.94	Ser: 6 Thr: 11 Tyr: 5	plasma
NIP	Rw2G029520.1	308	31977.05	8.35	Ser: 14 Thr: 12 Tyr: 1	plasma
	Rw2G030270.1	129	14276.47	9.34	Ser: 6 Thr: 2 Tyr: 1	plasma
	Rw2G041770.1	273	28748.53	10.56	Ser: 8 Thr: 4 Tyr: 1	plasma
	Rw2G050020.1	275	28658.46	8.43	Ser: 19 Thr: 5 Tyr: 0	cacuole
	Rw2G050030.1	262	27983.39	9.23	Ser: 12 Thr: 9 Tyr: 1	plasma
	Rw4G018020.1	305	32122.69	7.03	Ser: 12 Thr: 5 Tyr: 1	plasma
	Rw4G033150.1	283	30251.91	9.4	Ser: 19 Thr: 11 Tyr: 3	cacuole
	Rw5G008080.1	271	28892.56	8.34	Ser: 17 Thr: 9 Tyr: 2	plasma
	Rw5G008090.1	271	28954.73	9.17	Ser: 12 Thr: 10 Tyr: 2	plasma
	Rw6G020350.1	271	28981.62	8.91	Ser: 14 Thr: 10 Tyr: 2	plasma
	Rw7G008050.1	279	29048.62	8.63	Ser: 14 Thr: 2 Tyr: 5	cacuole
	Rw7G039920.1	231	24804.89	6.02	Ser: 16 Thr: 5 Tyr: 4	plasma
TIP	Rw0G004640.1	253	26078.94	5.57	Ser: 6 Thr: 4 Tyr: 4	plasma
	Rw1G029610.1	252	25893.92	5.7	Ser: 11 Thr: 4 Tyr: 1	plasma
	Rw1G034320.1	252	25806.17	6.01	Ser: 8 Thr: 6 Tyr: 2	plasma
	Rw2G033020.1	248	25493.72	5.61	Ser: 6 Thr: 2 Tyr: 3	plasma
	Rw2G033280.1	248	25493.72	5.61	Ser: 6 Thr: 2 Tyr: 3	plasma
	Rw3G015260.1	233	25058.94	9.92	Ser: 9 Thr: 6 Tyr: 2	cytosol
	Rw5G038800.1	254	26114.31	4.72	Ser: 11 Thr: 3 Tyr: 2	cacuole
	Rw6G040020.1	249	26438.7	5.8	Ser: 8 Thr: 4 Tyr: 1	cacuole
	Rw7G000060.1	259	27708.11	6.75	Ser: 9 Thr: 6 Tyr: 4	plasma
	Rw7G018050.1	249	25188.18	4.73	Ser: 14 Thr: 10 Tyr: 3	cacuole
SIP	Rw0G011440.1	239	25407.1	9.05	Ser: 6 Thr: 4 Tyr: 3	cacuole
	Rw2G023690.1	232	25343.09	9.49	Ser: 9 Thr: 7 Tyr: 0	cacuole
XIP	Rw6G002590.1	317	34073.15	7.73	Ser: 13 Thr: 6 Tyr: 1	cacuole
	Rw6G002600.1	318	34278.51	8.52	Ser: 12 Thr: 7 Tyr: 1	plasma
	Rw6G002610.1	235	25073.12	8.33	Ser: 9 Thr: 8 Tyr: 3	plasma

Table S2. Protein structure, phosphorylation sites and subcellular localization information for 38 RcAQPs.

	Sequence ID	Number of AA	MW	PI	No. of Phosphor Sites	Prediction for cellular-localization
PIP	RchChr1g0355771	287	30859.69	8.47	Ser: 5 Thr: 8 Tyr: 4	plasma
	RchChr1g0363361	283	30153.99	8.44	Ser: 16 Thr: 9 Tyr: 3	plasma
	RchChr1g0369541	290	31107.29	8.9	Ser: 8 Thr: 10 Tyr: 3	plasma
	RchChr3g0469531	284	30006.71	8.6	Ser: 11 Thr: 8 Tyr: 3	plasma
	RchChr3g0469541	284	30031.84	8.28	Ser: 10 Thr: 8 Tyr: 3	plasma
	RchChr5g0059051	281	30195.11	7.05	Ser: 10 Thr: 7 Tyr: 4	plasma
	RchChr6g0278401	280	29964.85	8.99	Ser: 10 Thr: 5 Tyr: 3	plasma
	RchChr6g0303511	283	30007.65	6.04	Ser: 12 Thr: 6 Tyr: 4	plasma
	RchChr6g0303531	148	16017.4	9.25	Ser: 7 Thr: 4 Tyr: 4	cacuole
	RchChr7g0197451	286	30903.97	8.94	Ser: 6 Thr: 12 Tyr: 5	plasma
NIP	RchChr2g0133211	308	31993.01	8.36	Ser: 14 Thr: 13 Tyr: 1	plasma
	RchChr2g0154411	102	10837.75	9.03	Ser: 4 Thr: 1 Tyr: 1	cacuole
	RchChr2g0154421	139	14610.84	8.93	Ser: 4 Thr: 4 Tyr: 0	plasma
	RchChr2g0168391	461	48311.43	9.31	Ser: 27 Thr: 8 Tyr: 1	plasma
	RchChr4g0417871	292	30511.67	6.63	Ser: 12 Thr: 5 Tyr: 2	plasma
	RchChr4g0441301	283	30191.83	9.38	Ser: 19 Thr: 11 Tyr: 3	cacuole
	RchChr5g0012031	286	30398.39	8.74	Ser: 24 Thr: 9 Tyr: 2	plasma
	RchChr5g0012041	271	29051.83	9.27	Ser: 11 Thr: 10 Tyr: 2	plasma
	RchChr6g0279421	271	28992.68	8.91	Ser: 14 Thr: 10 Tyr: 2	plasma
	RchChr7g0188201	301	31274.18	8.48	Ser: 13 Thr: 10 Tyr: 3	cacuole
	RchChr7g0238841	232	26009.1	8.26	Ser: 14 Thr: 7 Tyr: 2	plasma
TIP	RchChr1g0365831	252	25893.92	5.7	Ser: 11 Thr: 4 Tyr: 1	plasma
	RchChr1g0374221	252	25790.17	6.01	Ser: 8 Thr: 6 Tyr: 2	plasma
	RchChr2g0139491	316	33467.08	5.2	Ser: 11 Thr: 2 Tyr: 3	cacuole
	RchChr3g0470701	253	26089.96	5.57	Ser: 3 Thr: 3 Tyr: 4	plasma
	RchChr5g0062791	254	26128.34	4.72	Ser: 11 Thr: 3 Tyr: 2	cacuole
	RchChr6g0306101	249	26439.68	5.59	Ser: 8 Thr: 4 Tyr: 2	cacuole
	RchChr7g0176871	259	27696.11	6.75	Ser: 8 Thr: 5 Tyr: 4	plasma
	RchChr7g0203581	249	25198.22	4.73	Ser: 13 Thr: 2 Tyr: 4	cacuole
SIP	RchChr2g0122531	232	25422.21	9.61	Ser: 9 Thr: 6 Tyr: 0	plasma
	RchChr2g0159011	241	25486.29	9.96	Ser: 11 Thr: 6 Tyr: 2	plasma
	RchChr3g0497551	239	25377.03	9.05	Ser: 7 Thr: 3 Tyr: 3	cacuole
XIP	RchChr6g0245741	235	24210.59	5.81	Ser: 18 Thr: 5 Tyr: 1	cacuole
	RchChr6g0245821	222	24327.86	9.03	Ser: 7 Thr: 3 Tyr: 3	plasma
	RchChr6g0245841	317	34125.22	8.27	Ser: 13 Thr: 6 Tyr: 1	cacuole
	RchChr6g0245851	254	27254.09	7.01	Ser: 13 Thr: 8 Tyr: 2	plasma
	RchChr6g0245861	318	34269.5	8.21	Ser: 12 Thr: 7 Tyr: 1	plasma
	RchChr6g0245871	284	30533.25	7.57	Ser: 14 Thr: 9 Tyr: 3	plasma

Table S3. Selection pressure (*Ka/Ks*) analysis for 34 ortholog pairs of AQPs between BT and OB.

Gene pair	Ka	Ks	Ka/Ks
Rw0G004180.1/RchChr2g0098701	0.21908648	0.298002889	0.735182405
Rw1G023010.1/RchChr1g0355771	0.001549587	0.023624	0.065593774
Rw1G027940.1/RchChr1g0363361	0	0.009324129	0
Rw1G029610.1/RchChr1g0365831	0	0.015734843	0
Rw1G031660.1/RchChr1g0369541	0.003041444	0.038890851	0.078204609
Rw1G034320.1/RchChr1g0374221	0.001798023	0.025534381	0.070415767
Rw2G023690.1/RchChr2g0122531	0.007650688	0.011822905	0.647107289
Rw2G029520.1/RchChr2g0133211	0.002921133	0.008450794	0.345663771
Rw2G033020.1/RchChr2g0139491	0	0.005217412	0
Rw2G033280.1/RchChr2g0139491	0	0.005217412	0
Rw2G041770.1/RchChr2g0154421	0.111966071	0.188139721	0.595121915
Rw2G050020.1/RchChr2g0168391	0.126792894	0.399733821	0.317193309
Rw2G050030.1/RchChr2g0168391	0.097367795	0.118963884	0.818465167
Rw3G014300.1/RchChr3g0469531	0.003142188	0.009397147	0.334376843
Rw3G014320.1/RchChr3g0469541	0	0.014162498	0
Rw3G015260.1/RchChr3g0470701	0.059688858	0.144112035	0.414183714
Rw4G018020.1/RchChr4g0417871	0.015298715	0.023463064	0.652034004
Rw4G033150.1/RchChr4g0441301	0.003145483	0.023982859	0.131155465
Rw5G008080.1/RchChr5g0012041	0.050852571	0.196095608	0.259325395
Rw5G008090.1/RchChr5g0012041	0.003292638	0.014833611	0.221971444
Rw5G036290.1/RchChr5g0059051	0	0.024372575	0
Rw5G038800.1/RchChr5g0062791	0.001777779	0.010126736	0.175552971
Rw6G002590.1/RchChr6g0245841	0.005630525	0.061280881	0.091880614
Rw6G002600.1/RchChr6g0245861	0.008450794	0.029748942	0.28407039
Rw6G002610.1/RchChr6g0245871	0	0.023257678	0
Rw6G019710.1/RchChr6g0278401	0.003198299	0.048403891	0.066075248
Rw6G020350.1/RchChr6g0279421	0.009852358	0.030616496	0.321799022
Rw6G037770.1/RchChr6g0303511	0.010016843	0.029129875	0.343868396
Rw6G040020.1/RchChr6g0306101	0.001804512	0.03189143	0.056582981
Rw7G000060.1/RchChr7g0176871	0.003479276	0.035690676	0.097484161
Rw7G008050.1/RchChr7g0188201	0.001645188	0.035854474	0.045885165
Rw7G014370.1/RchChr7g0197451	0.004666855	0.019003392	0.245580083
Rw7G018050.1/RchChr7g0203581	0.001832062	0.020220264	0.090605244
Rw7G039920.1/RchChr7g0238841	0.419028579	0.481145196	0.870898395

Table S4. Primers used in this study.

	Primers name	Sequence (5'-3')
Primers for constructs in VIGS	FRcPIP2A	CTCCATGGGGATCCGACAGAGCCATCATTGCAGAA
	RRcPIP2A	AGACGCGTGAGCTCGCCAAGGATTGAGCCACAATA
	JC-F	TGGGAGATGATACGCTGTT
	JC-R	CCTAAAACTTCAGACACG
	CXTRV2F	GTTACTCAAGGAAGCACG
	CXTRV2R	ACCGTAGTTTAATGTCTTCG
Primers for qPCR	qPCR-PIP2-F	GAAGTTTCGGAGCTGCAGTG
	qPCR-PIP2-R	AAAGAACCCGAAGCCTTAGCA
	qPCR-UBC-F	GCCAGAGATTGCCCATATGTA
	qPCR-UBC-R	TCACAGAGTCCTAGCAGCACA
